# Polycaprolactone scaffolds promote a regeneration-supportive metabolic response attenuated in diabetes

**DOI:** 10.1093/rb/rbag177

**Published:** 2026-08-17

**Authors:** Wing Lee Chan, Raphaela Fritsche-Guenther, André Bembennek, Agnes Ellinghaus, Jan Baumbach, Georg N Duda, Jennifer Kirwan, Tanja Laske, Patrina S P Poh

**Affiliations:** Julius Wolff Institute, Berlin Institute of Health at Charité-Universitätsmedizin Berlin, Berlin 13353, Germany; Berlin Institute of Health at Charité-BIH Metabolomics, Berlin 10117, Germany; Institute for Computational Systems Biomedicine, University of Hamburg, Hamburg 22761, Germany; Viral Systems Modeling, Leibniz Institute of Virology, Hamburg 20251, Germany; Julius Wolff Institute, Berlin Institute of Health at Charité-Universitätsmedizin Berlin, Berlin 13353, Germany; Institute for Computational Systems Biomedicine, University of Hamburg, Hamburg 22761, Germany; Computational Biomedicine Lab, Department of Mathematics and Computer Science, University of Southern Denmark, Odense 5230, Denmark; Julius Wolff Institute, Berlin Institute of Health at Charité-Universitätsmedizin Berlin, Berlin 13353, Germany; Berlin Institute of Health at Charité-BIH Metabolomics, Berlin 10117, Germany; Institute for Computational Systems Biomedicine, University of Hamburg, Hamburg 22761, Germany; Viral Systems Modeling, Leibniz Institute of Virology, Hamburg 20251, Germany; Julius Wolff Institute, Berlin Institute of Health at Charité-Universitätsmedizin Berlin, Berlin 13353, Germany

**Keywords:** biomaterial–host interaction, metabolomics, type 2 diabetes, scaffold-guided bone regeneration, polycaprolactone

## Abstract

Scaffold-guided bone regeneration (SGBR) is a promising strategy for repairing large bone defects. However, its efficacy is markedly reduced in diabetes. Here, we used non-diabetic and diabetic Zucker Diabetic Fatty (ZDF) rats with sham-operated, empty-defect and scaffold-treated groups to examine how host metabolic state influences the regenerative performance of 3D-printed polycaprolactone (PCL) scaffolds in critical-sized femoral defects. Targeted metabolomics of plasma and femoral midshaft revealed a distinct diabetic signature that was characterized by carbohydrate accumulation and broad amino acid dysregulation. In non-diabetic rats, PCL scaffolds enhanced bone regeneration and were associated with a local metabolic response consistent with a regeneration-supportive increase in energy and substrate availability, including glutamate-related metabolism. In diabetic rats, empty defects showed elevated branched-chain amino acids (BCAAs) and reduced glutamine, together with limited bone regeneration. Although scaffold treatment partially normalized BCAAs in diabetic rats, glutamate was not restored and the regenerative benefit of scaffold implantation was lost. These data indicate that SGBR is associated with a regeneration-supportive metabolic response in non-diabetic bone defects, whereas this response is blunted in the diabetic host. This study underscores the need for biomaterial strategies better matched to metabolically compromised host conditions, such as diabetes, to enable effective scaffold-guided regeneration.

## Graphical abstract

**Graphical Abstract:**
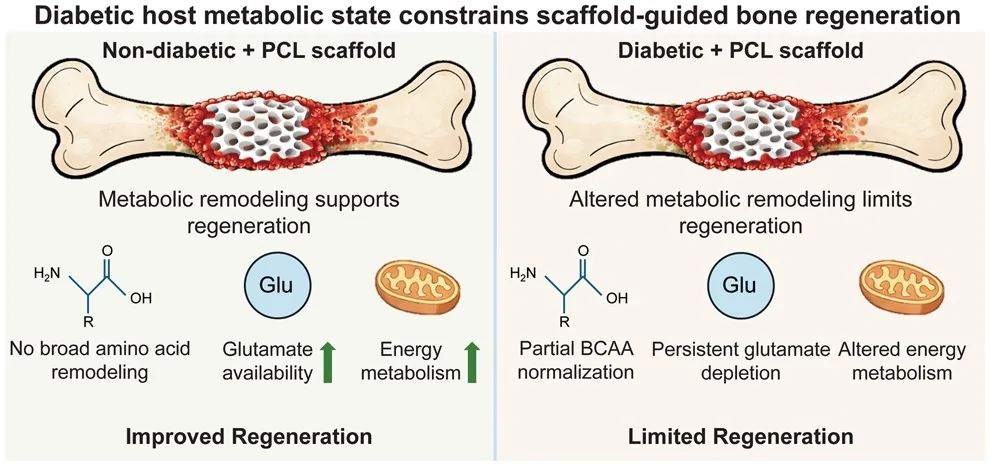

## Introduction

Diabetes mellitus is a chronic metabolic disease characterized by impaired insulin action and sustained hyperglycemia. According to projections by the International Diabetes Federation (IDF), approximately 1 in 8 adults (∼783 million individuals) will have diabetes by 2045, with over 90% likely affected by type 2 diabetes (T2D) [1]. Diabetes is associated with reduced bone quality, delayed fracture repair and impaired regeneration capacity. Despite these deficits, clinical management of large bone defects remains similar for diabetic and non-diabetic patients and relies on graft-based approaches such as autografts, allografts, vascularized fibular grafts, hybrid grafts, the Masquelet technique, distraction osteogenesis or segmental prostheses [2]. These treatments do not address the metabolic limitations of the diabetic host.

Scaffold-guided bone regeneration (SGBR) offers an alternative strategy for large defect repair. In this context, scaffolds act as 3D biomaterial templates that maintain space, provide structural guidance and support tissue ingrowth during defect bridging [3–5]. Recent scaffold development has extended this structural principle by incorporating bioactive or stimuli-responsive functions designed to modulate the local repair microenvironment [6]. However, how diabetes modifies the biochemical response to a scaffold *in vivo* remains unclear [7, 8]. As a result, scaffold performance may depend not only on architectural features but also on whether the host can support the energy and biosynthetic demands of regeneration. Our previous study indicated that delayed SGBR in the LundMetS diabetic rat model is associated with disrupted energy metabolism and impaired amino acid synthesis impacting collagen formation [9]. However, it remained unclear how scaffold implantation reshapes the regenerative metabolome relative to baseline host metabolism and defect-associated changes, and how this scaffold-associated metabolic response is altered in the diabetic host across systemic and local compartments. Clarifying this relationship is important for understanding how host physiology influences scaffold function *in vivo* and for guiding biomaterial strategies better matched to metabolically compromised conditions.

In the current study, the Zucker Diabetic Fatty (ZDF) rat model, which is characterized by a spontaneous mutation in the leptin receptor gene (Lepr; fa allele), was used. This mutation results in insulin resistance, hyperglycemia, hyperlipidemia and metabolic dysfunction similar to human T2D. Unlike LundMetS rats that develop T2D-like symptoms on a standard diet, ZDF rats require dietary induction to develop overt diabetes. The use of this distinct diet-induced diabetic model, together with sham-operated, empty-defect and scaffold-treated groups, complements the previous LundMetS study and allows the effect of scaffold implantation on the regenerative metabolome to be distinguished from baseline host metabolism and defect-associated metabolic responses during bone healing.

In this study, we investigated how host metabolic state shapes scaffold-guided bone regeneration using the Zucker Diabetic Fatty (ZDF) rat model of type 2 diabetes. We hypothesized that SGBR is associated with a regeneration-supportive metabolic response in non-diabetic defects, and that this scaffold-associated response is blunted in diabetic bone defects. To test this hypothesis, metabolomic profiles of plasma and regenerating bone were analyzed in diabetic and non-diabetic animals, and metabolic features associated with sham-operated, untreated and polycaprolactone (PCL) scaffold-treated defects were compared. By linking systemic and local metabolic states to regenerative outcomes, this study aims to clarify how host metabolism constrains scaffold function in a metabolically compromised setting.

## Materials and methods

### Scaffold manufacturing and characterization

Gyroid‐architecture scaffolds with 69% porosity were designed in Rhino3D. A custom-made pneumatic melt-extrusion 3D printer was then used to fabricate the scaffolds from medical-grade 50-kDa PCL (Evonik) at 100°C, 0.15 kPa air pressure and a print speed of 200 mm/min. Initial blocks (12 × 6 × 6 mm) were biopsy-punched into Ø 4 mm cylinders. Prior to use, all scaffolds were treated with 100% oxygen plasma (25 min, 0.3 mbar, 30 W).

Scaffold architecture was assessed by microcomputed tomography (µCT; SkyScan 1272, Bruker) to quantify porosity, wall thickness and pore size distribution. Six scaffolds were scanned at 70 kV and 142 µA using a 500 µm aluminum filter and 10 µm voxel resolution. Image reconstruction was performed with GPUReconServer (v2.1.0.0), and the resulting datasets were analyzed in CT Analyser (v1.20.8.0) using an adaptive gray scale threshold range of 40–255. A 3-mm-long volume of interest was defined, and built-in CTAn macros were used to calculate total porosity, open porosity, closed porosity, wall thickness and pore-diameter distributions.

### *In vivo* study

This study was approved by LaGeSo Berlin (Ethics Approval No. G0296/19). Twelve-week-old male ZDF rats (Charles River Laboratories) with *ad libitum* access to Purina 5008 chow (Brogaarden, Denmark) and water were studied. Lean ZDF (fa/+) rats (*N* = 21), which remain metabolically normal, served as non-diabetic controls, whereas obese ZDF (fa/fa) rats (*N* = 21), which exhibit severe obesity and glucose intolerance, comprised the diabetic group (Supplementary Figure S1). The animals were divided into experimental groups as listed in Table 1, with each treatment group represented in both non-diabetic and diabetic conditions for intra- and intergroup comparisons (Sham: *N* = 5 per condition; Empty: *N* = 9 per condition; Scaffold: *N* = 7 per condition). Final metabolomics sample sizes after sample availability and GC–MS quality control are reported in the corresponding Supplementary Tables.

**Table 1:** *In vivo* study experimental groups for both non-diabetic and diabetic animals.

Sham group	
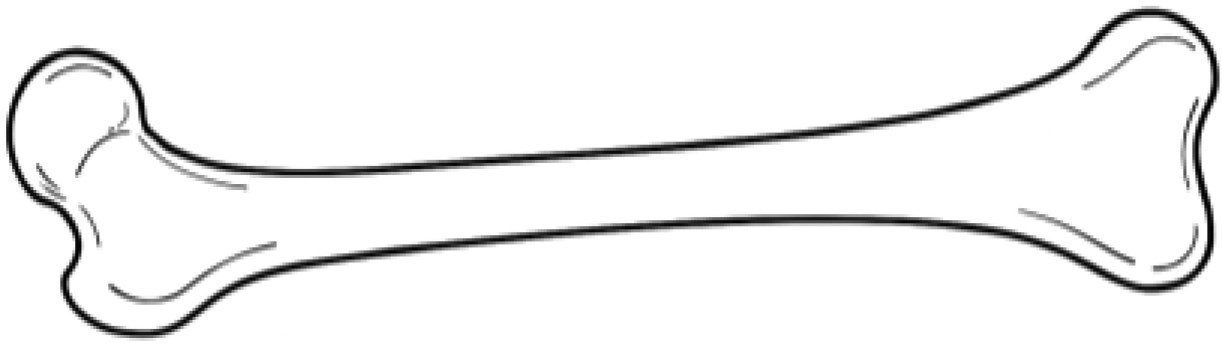	Animals received anesthesia only.
**Empty group**	
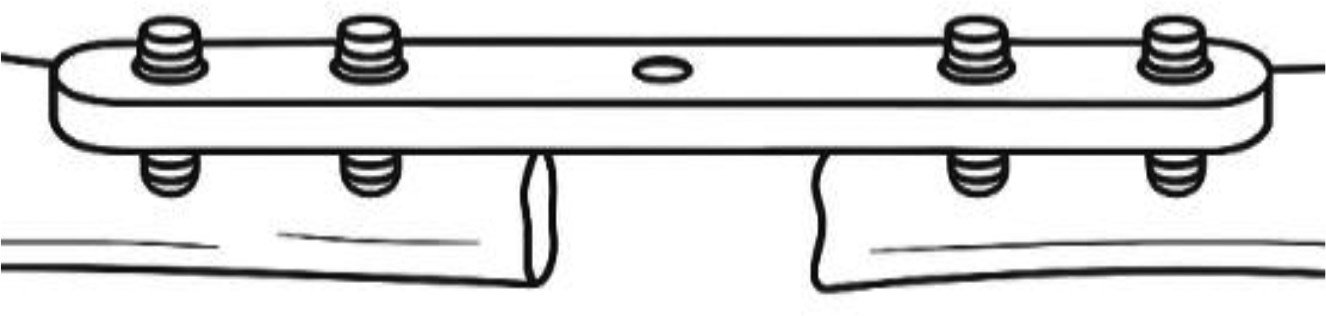	Animals were anesthetized, and a 5-mm segmental femoral defect was created and left empty.
**Scaffold group**	
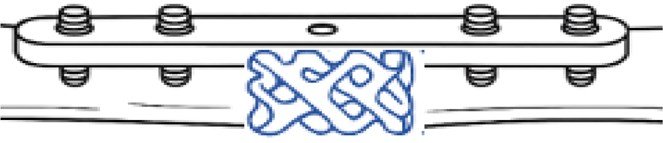	Animals were anesthetized, and a 5-mm segmental femoral defect was created and fitted with a gyroid-architecture scaffold.

Each treatment group was represented in both host conditions to enable intra- and intergroup comparisons.

All procedures complied with the Animal Welfare Act and national guidelines. Under isoflurane anesthesia (Forene; AbbVie Deutschland GmbH) and buprenorphine analgesia (0.03 mg/kg body weight (BW); Essex Pharma), a 5-mm critical-sized defect was created in the left femoral midshaft of the rat. The femur was exposed via an anterolateral approach, stabilized with a polyetheretherketone (PEEK) plate secured by two proximal and two distal stainless steel pins (RISystem, Switzerland), and the defect was left empty or bridged by a scaffold. Muscle and skin were closed, and post-operative analgesia (tramadol, 0.5 mg/mL in drinking water) was provided for three days. Rats resumed unrestricted activity, food and water immediately after operation. At the experimental endpoint, 6 weeks post-operation, blood glucose concentration was measured using CONTOUR NEXT (Ascensia, Germany). The whole femur and tibia were explanted. Whole blood (500 µL) was collected in a BD Microtainer™ tube containing K2EDTA (Fisher Scientific, Germany). Samples were centrifuged at 1000 × g for 10 min to isolate plasma, which was stored at −80°C until GC–MS analysis.

### µCT of bone explants

The whole femur and tibia explants were scanned *ex vivo* by µCT (U-CTHR, MILabs) in fast mode (3 min) to minimize tissue damage, using a 50 kV/0.21 mA X-ray beam with a 500 µm Al filter, 360° rotation and 10 µm voxels. Images were reconstructed in MILabs RECON and aligned in DataViewer (Bruker, MA, USA). Quantitative analysis was performed using CTAn (Bruker, MA, USA). A volume of interest (VOI) was defined to include the 5 mm defect region and extend 0.5 mm proximally and distally from each defect cutting plane. Immediately after µCT, the explants were snap-frozen in liquid nitrogen for metabolomics.

### Metabolomics by gas chromatography-mass spectrometry (GC–MS)

GC-MS-based metabolomics was used to analyze bone and plasma samples. Prior to analysis, a customized jig was used to stabilize bone and allow retrieval of the midshaft or healing bone defect region in a standardized way (5 mm plus 1 mm on each extremity of the defect). Then, samples were homogenized using an automated pulveriser (CP02 cryoPREP® Covaris), transferred into new Eppendorf vials and weighed.

#### Metabolite extraction and derivatization

Metabolites from plasma and homogenized bone samples were extracted using the Fiehn method [10]. Briefly, metabolites were extracted by adding 1 mL of extraction buffer per 30 µL of plasma, or 1 mL of extraction buffer per 50 mg of homogenized bone tissue. The extraction buffer consisted of acetonitrile: isopropanol: water (3:3:2, v/v/v) and was added directly to the plasma or bone samples, which were vortexed and shaken for 5 min at 4°C at 1200 rpm (Thermoshaker) followed by centrifugation for 2 min at 9000 × g at 4°C. The supernatant was vacuum-dried. The samples were then re-suspended in 450 µL acetonitrile: water (50:50, v/v) at room temperature, vortexed for 10 s and centrifuged for 2 min at 14 000 × g at room temperature. Two aliquots of 200 µL of the supernatant were vacuum-dried and stored at −80°C until downstream metabolomics analysis.

Pooled quality control (QC) samples were generated by combining aliquots from all plasma or bone samples. Derivatization was performed in two steps using methoxyamine in pyridine followed by N-methyl-N-(trimethylsilyl)trifluoroacetamide (MSTFA). A QC mix and a fatty acid methyl ester (FAME) mixture were prepared alongside the explanted samples using the same procedure. The QC mix, prepared in-house from commercial standards, was used for metabolite identification, while the FAME mixture (identical to that used for retention index calibration) also served as a wash solution to monitor potential carryover or system contamination.

#### GC–MS for central carbon metabolites

The metabolomics screening was conducted using a Pegasus BT GCxGC-TOF-MS system (LECO Corporation, St. Joseph, MI, USA) equipped with a Gerstel auto-sampler. Chromatographic separation was achieved using an Agilent 8890 GC (Agilent Technologies, Santa Clara, CA, USA) fitted with an RTX-5 MS column with a 10 m integra-guard (30 m × 250 µm i.d., 0.25 µm film thickness; Restek, Fuldabrück, Germany). Helium was used as the carrier gas at a constant flow rate of 1 mL/min. The inlet temperature was set to 200°C, and separation was performed using the following oven temperature program: 50°C for 1 min, ramped at 20°C/min to 330°C, held for 5 min. Spectra were acquired over a mass range of m/z 60–550 at 10 spectra/s in splitless mode.

#### GC–MS data processing and statistical analysis

Data acquisition and processing were performed using ChromaTOF software (LECO), which included baseline correction, peak detection and quantification of peak area and height. Identification was based on an internal reference library comprising 67 metabolites (with 72 derivatives), developed in-house. QC samples were analyzed independently to assess system performance and data consistency [11]. The analysis initially detected 40 metabolites.

#### Scaling

To scale each compound relative to the corresponding sham baseline, metabolite values were first log-transformed. For each compound, the median log-transformed value was then calculated from the sham samples. For empty-defect and scaffold-treated samples, this compound-specific sham median was subtracted from the corresponding log-transformed metabolite value. This procedure was performed separately for diabetic and non-diabetic animals, so that defect- and scaffold-associated changes were evaluated relative to the appropriate host-state-specific sham baseline (Supplementary Figures S2 and S3). This scaling was used to reduce the influence of baseline metabolic differences between diabetic and non-diabetic animals and to distinguish treatment-associated metabolic deviations from pre-existing host-state effects. After scaling, clustering of the molecular profiles by disease and treatment context improved (Supplementary Figure S2).

#### Identification of disease-associated metabolic indicators

Univariate receiver operating characteristic (ROC) analysis was conducted using the MetaboAnalystR 4.0 package as well as the web version of MetaboAnalyst 6.0 to evaluate the discriminatory power of individual metabolites. Classification performance was quantified using the Area Under the Curve (AUC), which reflects the trade-off between sensitivity (true positive rate) and specificity (1 − false positive rate). For sham baseline comparisons (Figure 1), unpaired two-sided Student’s *t*-tests were performed for each compound using raw metabolite abundance values. For empty-defect and scaffold-treated comparisons, unpaired two-sided Student’s *t*-tests were performed for each compound using sham-scaled log-transformed values. Limma differential-abundance analysis was additionally performed using the R package limma. To address multiple testing, raw *P*-values were adjusted using the Benjamini–Hochberg false discovery rate procedure within predefined biological comparison families, separately for each compartment and contrast. All detected metabolites with valid *P*-values were included in each correction family. Metabolites with *q* < 0.05 were considered FDR-significant, whereas metabolites with nominal *P* < 0.05 but *q* ≥ 0.05 were interpreted as exploratory findings.

**Figure 1:**
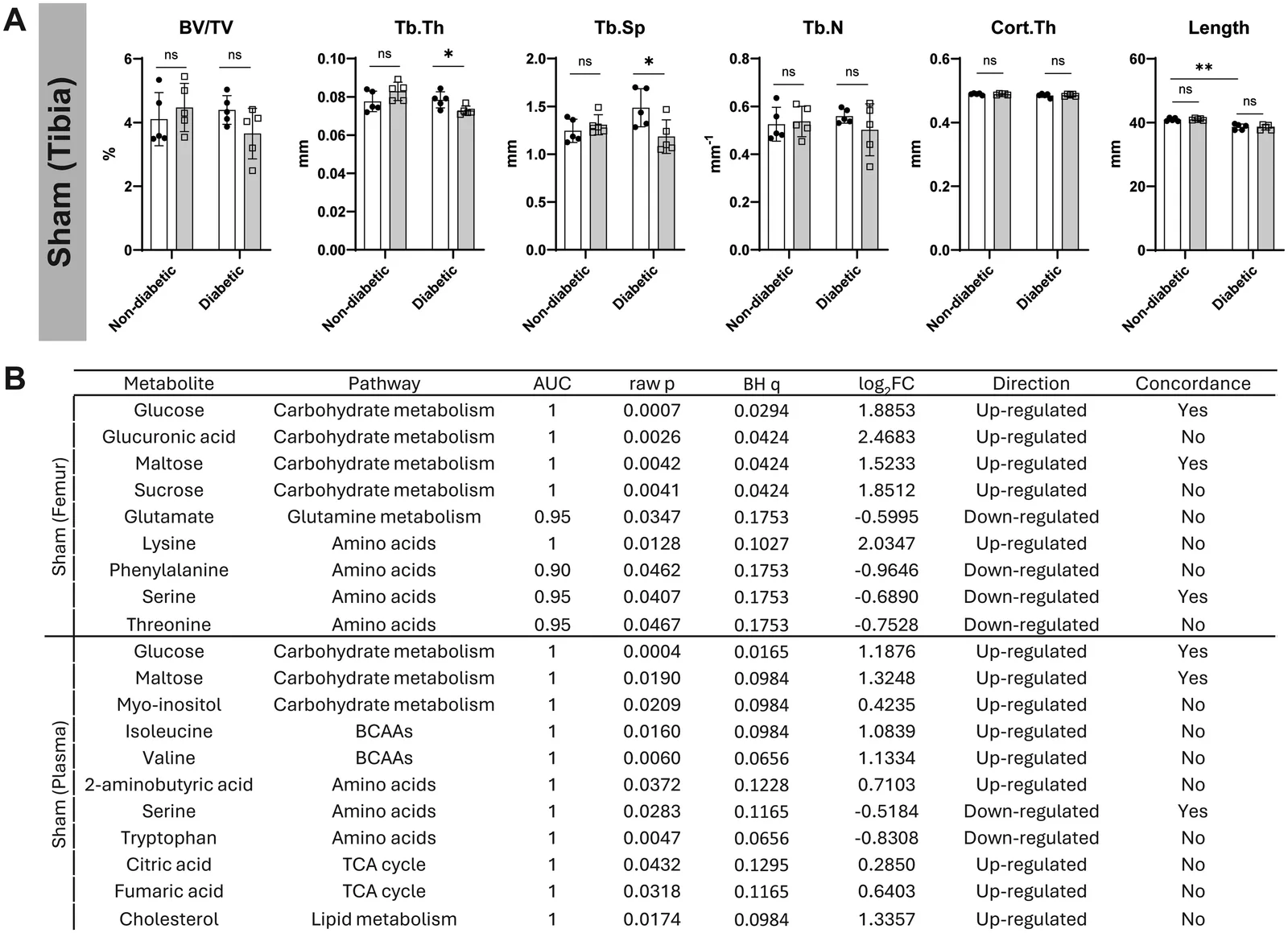
Bone morphology and metabolic profile of sham animals. (**A**) Quantitative micro-computed tomography (μCT) analysis of tibial trabecular architecture in non-diabetic and diabetic rats: bone volume fraction (BV/TV), trabecular number (Tb.N), trabecular thickness (Tb.Th), trabecular separation (Tb.Sp), cortical thickness (Cort.Th) and length. White bars represent the left tibia and light grey bars represent the right tibia. Data are mean ± SD. Statistical comparisons were performed using unpaired two-sided Welch’s *t*-tests. ns, not significant; *P < 0.05; **P < 0.01. (**B**) Metabolites with strong discriminatory power (AUC ≥ 0.9, raw *P* < 0.05) identified in the femoral midshaft and plasma from diabetic and non-diabetic rats. AUC values indicate univariate receiver operating characteristic performance. Raw *P*-values were calculated using unpaired two-sided Student’s *t*-tests, and Benjamini–Hochberg *q*-values were calculated across all tested metabolites within each compartment. log₂FC was calculated as diabetic relative to non-diabetic sham animals; positive values indicate higher abundance in diabetic animals and negative values indicate lower abundance. ‘Direction’ indicates whether each metabolite was increased or decreased in diabetic samples, and ‘Concordance’ shows whether the regulation pattern was consistent across compartments.

### Declaration of generative AI and AI-assisted technologies in the manuscript preparation process

During the preparation of this work, the authors used ChatGPT (OpenAI) to assist with language editing and improvement of text clarity. After using this tool, the authors reviewed and edited the content as needed and take full responsibility for the content of the published article.

## Results and discussion

### Diabetic ZDF rats show limited baseline skeletal differences but a distinct bone and plasma metabolic state

At 18 weeks of age, ZDF diabetic rats exhibited significantly higher body weight compared to age-matched non-diabetic controls (408 ± 37 g vs 340 ± 24 g; *P* < 0.001; *n* = 25 per host condition). Blood glucose concentrations were also markedly elevated in diabetic rats (454 ± 108 mg/dL) relative to non-diabetic controls (144 ± 17 mg/dL; *P* < 0.001; *n* = 25 per host condition), confirming systemic hyperglycemia (Supplementary Figure S1). By contrast, quantitative μCT analysis of the tibia showed comparable bone volume fraction (BV/TV), trabecular number (Tb.N), trabecular thickness (Tb.Th) and trabecular separation (Tb.Sp) between diabetic and non-diabetic rats (Figure 1A). Minor side-to-side variations were observed in some diabetic animals, where the right tibia exhibited slightly lower Tb.Th and Tb.Sp relative to the left tibia, but this pattern was not consistent across animals. The tibiae of diabetic rats were slightly shorter than those of non-diabetic rats. These data indicate that the diabetic rats used in this study did not yet exhibit a pronounced baseline skeletal phenotype.

To establish the baseline metabolic differences independent of defect regeneration, we performed metabolomic profiling in sham-operated animals on (i) the midshaft of the left femur, which was the same anatomical region used for defect creation in the operated groups, and (ii) plasma. In bone, diabetic animals showed elevated levels of glucose, glucuronic acid, maltose, sucrose and lysine accompanied by reduced levels of glutamate, phenylalanine, serine and threonine (Figure 1B). At the systemic level, plasma of diabetic animals revealed elevated glucose, maltose, and myo-inositol, cholesterol, citric acid, fumaric acid, isoleucine, valine and 2-aminobutyric acid; while serine and tryptophan were decreased when compared to plasma of non-diabetic rats (Figure 1B). Complete metabolite-level statistics for the femoral midshaft and plasma sham comparisons are provided in Supplementary Tables S1 and S2. These data define a distinct diabetic baseline characterized by carbohydrate accumulation and amino acid dysregulation in both compartments. The absence of marked structural differences at this time point suggests that metabolic disruption precedes overt skeletal deterioration in this model.

Integration of bone and plasma metabolomics revealed three directionally consistent cross-compartment changes. Glucose and maltose were elevated in diabetic rats in both bone and plasma, whereas serine showed a concordant reduction. After Benjamini–Hochberg correction, the strongest statistical support was observed for sugar-related metabolites, with glucose showing the most robust cross-compartment evidence. These findings indicate that systemic metabolic imbalance is reflected in the local bone metabolic milieu (Figure 1B). Elevated levels of sugar-related metabolites such as glucose reflect the well-established metabolic dysfunction of diabetes, in which impaired glycolysis and mitochondrial activity are reported across multiple tissues [12–19]. Serine did not remain FDR-significant, but its concordant reduction across compartments remains biologically plausible because decreased plasma serine levels have been reported in diabetes [20, 21] and have been implicated in diabetic neuropathy [22, 23] and impaired homocysteine clearance [20]. Thus, serine is interpreted as a literature-supported exploratory feature of the diabetic metabolic state, with its relevance for skeletal tissue requiring further validation. Taken together, these data establish that diabetic ZDF rats enter the regenerative challenge from a metabolically distinct host state, and this baseline may influence healing and scaffold response.

### PCL scaffolds promote bone regeneration in non-diabetic but not diabetic rats

Critical-sized femoral defects in non-diabetic and diabetic rats were either left empty or filled with a 3D-printed gyroid scaffold. µCT analysis of reconstructed 3D scaffold datasets confirmed a measured total porosity of 79.20 ± 0.91%, above the 69% design target. Open porosity was essentially identical to total porosity (79.20 ± 0.91%), whereas closed porosity was negligible (0.04 ± 0.02%), consistent with a highly interconnected pore network. Wall thickness and pore diameter predominantly clustered between 210 and 290 µm (Supplementary Figure S4). The scaffold therefore provided a reproducible porous architecture for testing host-state-dependent regenerative performance.

After 6 weeks, *ex vivo* μCT (Figure 2A and B) showed that scaffold implantation significantly increased BV/TV in non-diabetic rats compared to empty defects, whereas no such bone ingrowth was detectable in diabetic animals. As expected, empty defects in diabetic rats exhibited significantly lower BV/TV than those in non-diabetic controls. Together, these data show that scaffold-associated bone formation occurs in non-diabetic but not diabetic host environments. This contrast frames the central question of the study, namely whether successful scaffold-guided regeneration is associated with a distinct metabolic response that fails to emerge under diabetic conditions.

**Figure 2:**
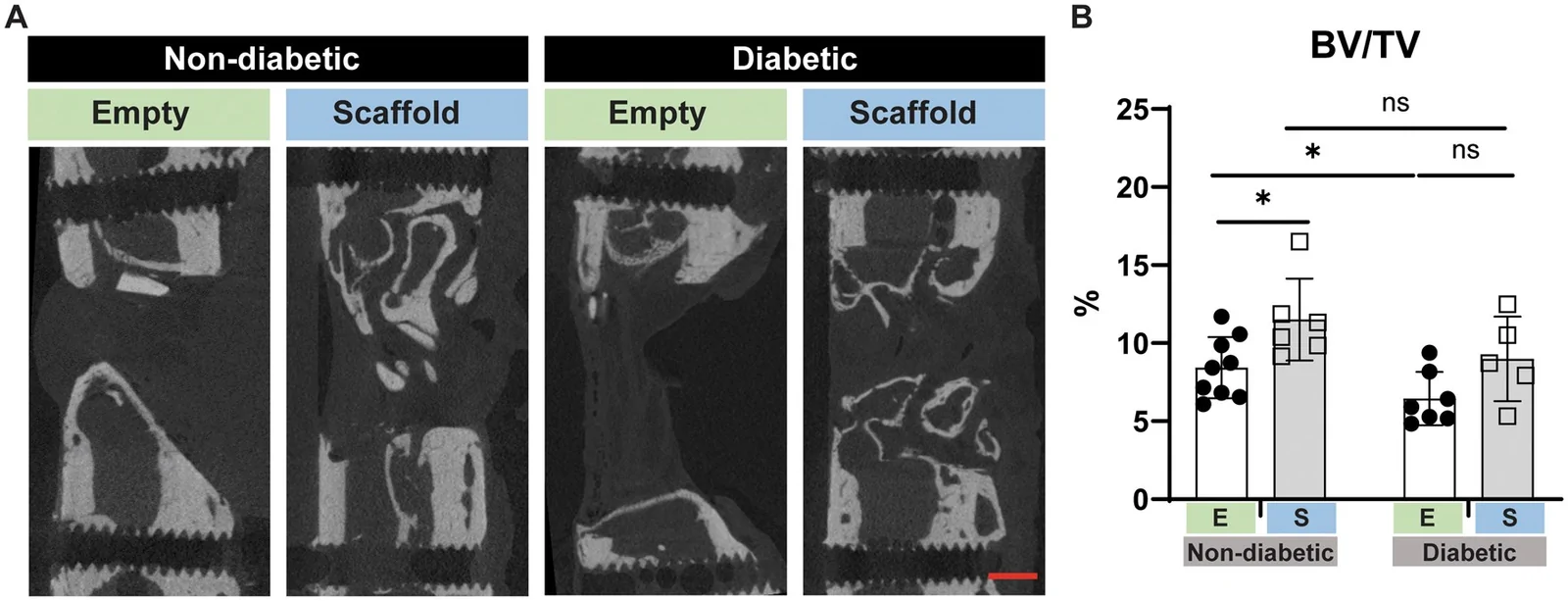
PCL scaffolds promote bone regeneration in non-diabetic but not diabetic rats. (**A**) Representative 3D μCT reconstructions of the femoral defect region, left empty or fitted with a PCL scaffold, in non-diabetic and diabetic rats at 6 weeks post-operation. Scale bar = 1 mm. (**B**) Bone volume/Tissue volume (BV/TV) (%) of newly formed bone within empty or scaffold-treated defects in non-diabetic versus diabetic rats at 6 weeks post-operation. Data are mean ± SD; *P < 0.05 by unpaired Welch’s *t*-test. E, empty defect; S, scaffold-treated defect. ns, not significant.

### Diabetes imposes a compartment-specific metabolic constraint during bone healing

Targeted metabolomics was performed on plasma and regenerating callus collected 6 weeks after surgery to define how diabetes alters the metabolic environment of bone healing at systemic and local levels (Figures 3 and 4). These profiles therefore define endpoint-associated metabolic states rather than the temporal sequence of metabolic remodeling during healing. Because host-state differences may depend on treatment context, diabetic and non-diabetic animals were compared separately in empty defects and scaffold-treated defects. This structure distinguishes the metabolic constraints of untreated healing from those that remain after scaffold implantation. Metabolites were evaluated using complementary ROC and limma differential-abundance analyses to identify features that discriminated between groups and to assess differential abundance, with ROC analysis providing a measure of discriminatory performance and limma differential-abundance providing statistical support for abundance differences (Supplementary Tables S3–S6).

**Figure 3:**
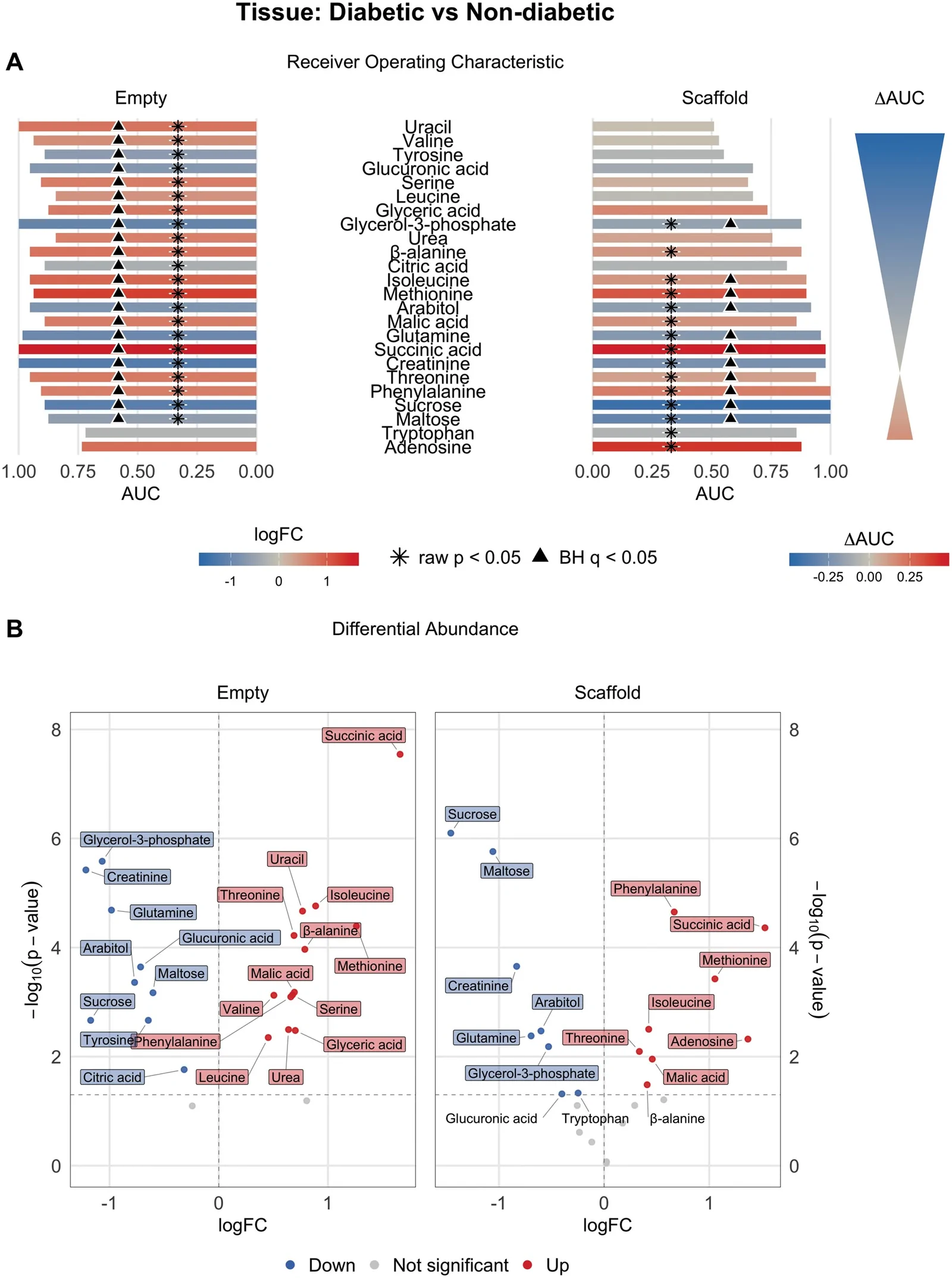
Metabolic alterations in diabetic vs non-diabetic animals in regenerating defects. (**A**) Horizontal bar plots show metabolites that discriminate between diabetic and non-diabetic animals when the bone defect was left empty (left, 8 diabetic samples and 8 non-diabetic samples) or treated with scaffold (right, 7 diabetic samples and 7 non-diabetic samples). Bars represent AUC values, with colors indicating log fold change (logFC) relative to the non-diabetic animals. Positive logFC (red) reflects increased metabolite abundance, while negative logFC (blue) indicates decreased abundance. Asterisks mark the treatment context in which the metabolite met the predefined ROC display criteria of AUC ≥ 0.8 and raw *P* < 0.05 (unadjusted *P*-values from an unpaired, two-sided Student’s *t*-test). Black triangles indicate metabolites that remained significant after Benjamini–Hochberg correction in the ROC analysis (BH *q* < 0.05). Metabolites are sorted by ΔAUC, the difference in AUC between scaffold-treated and empty-defect comparisons. (**B**) Volcano plots show limma differential-abundance analysis for the same comparisons. The *x*-axis represents logFC in diabetic animals relative to non-diabetic animals, and the *y*-axis represents − log₁₀ *P*-values. Compounds are only included in the volcano plot if they reach an AUC ≥ 0.8 in (A). Red and blue labels indicate metabolites with nominal limma *P* < 0.05 and higher or lower abundance in diabetic animals, respectively. Boxed labels indicate metabolites that remained significant after Benjamini–Hochberg correction in the limma differential-abundance analysis (BH *q* < 0.05).

**Figure 4:**
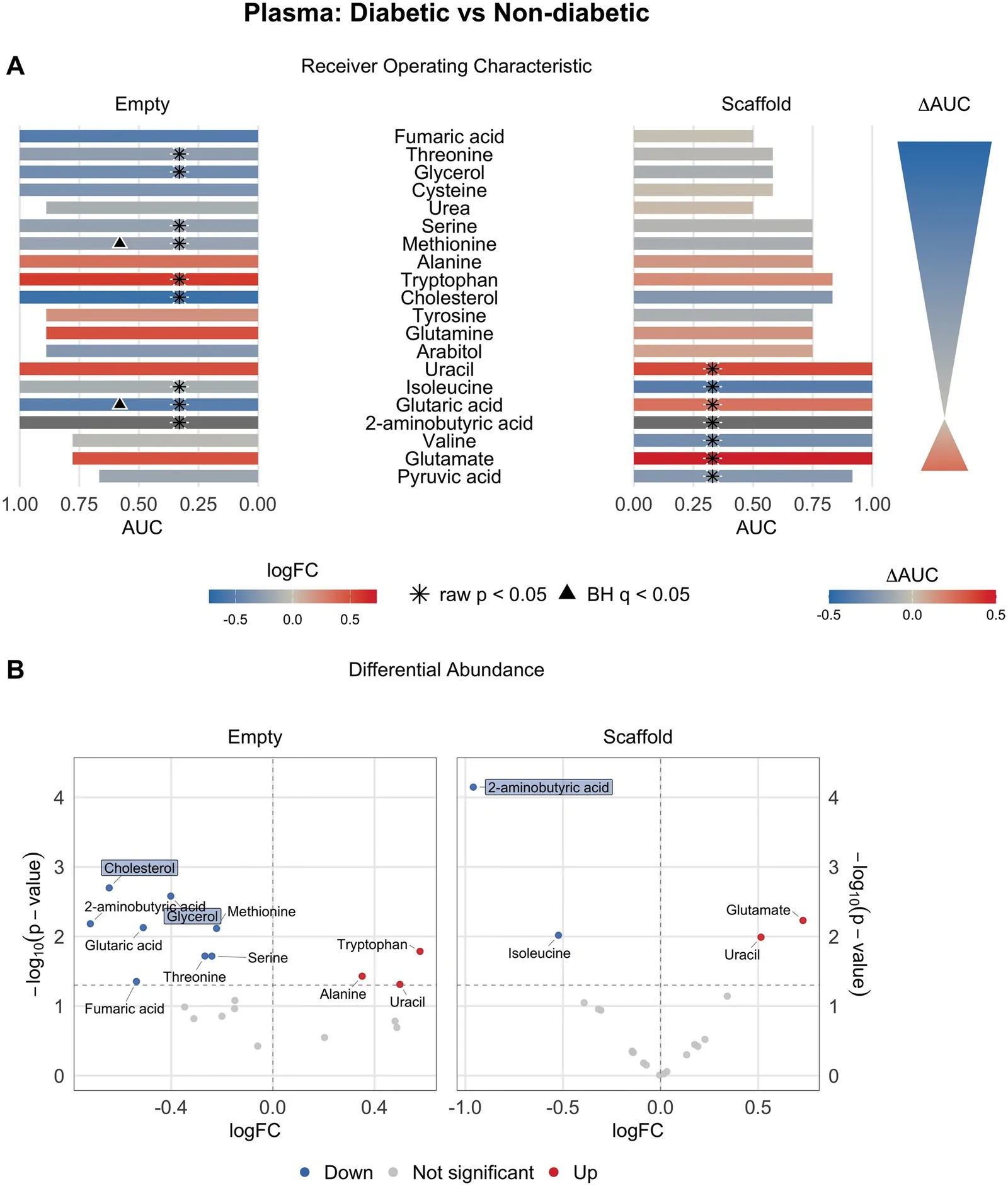
Metabolic alterations in diabetic vs non-diabetic animals in plasma samples. (**A**) Horizontal bar plots show metabolites that discriminate between diabetic and non-diabetic animals when the bone defect was left empty (left, 3 diabetic samples and 3 non-diabetic samples) or treated with scaffold (right, 4 diabetic samples and 3 non-diabetic samples). Bars represent AUC values, with colors indicating log fold change (logFC) relative to non-diabetic animals. Positive logFC (red) reflects increased metabolite abundance, while negative logFC (blue) indicates decreased abundance. Asterisks mark the treatment context in which the metabolite met the predefined ROC display criteria of AUC ≥ 0.8 and raw *P* < 0.05 (unadjusted *P*-values from an unpaired, two-sided Student’s *t*-test). Black triangles indicate metabolites that remained significant after Benjamini–Hochberg correction in the ROC analysis (BH *q* < 0.05). Metabolites are sorted by ΔAUC, the difference in AUC between scaffold-treated and empty-defect comparisons. (**B**) Volcano plots show limma differential-abundance analysis for the same comparisons. The *x*-axis represents logFC in diabetic animals relative to non-diabetic animals, and the *y*-axis represents − log₁₀ *P*-values. Compounds are only included in the volcano plot if they reach an AUC ≥ 0.8 in (A). Red and blue labels indicate metabolites with nominal limma *P* < 0.05 and higher or lower abundance in diabetic animals, respectively. Boxed labels indicate metabolites that remained significant after Benjamini–Hochberg correction in the limma differential-abundance analysis (BH *q* < 0.05).

#### Untreated (empty) bone defect

In regenerating tissue from empty defects, diabetes was associated with a strong local metabolic divergence. ROC analysis identified 22 metabolites that discriminated between diabetic and non-diabetic animals after Benjamini–Hochberg correction, and all 22 were also supported by limma differential-abundance analysis. Limma differential-abundance analysis additionally identified fructose-6-phosphate as FDR-significant (Figure 3A and B; Supplementary Table S3). Relative to non-diabetic callus, diabetic callus showed reduced maltose, sucrose, glucuronic acid, citric acid and glycerol-3-phosphate, together with increased glyceric acid, malic acid and succinic acid. Amino acid metabolism was also markedly altered, with increased isoleucine, leucine, valine, methionine, phenylalanine, threonine, β-alanine, and serine, and reduced glutamine and tyrosine. Additional differences included uracil, arabitol, urea and creatinine. Thus, untreated diabetic defects showed coordinated differences in carbohydrate-related metabolites, TCA-cycle-associated intermediates and amino acid metabolism.

This local pattern indicates that diabetes constrains the metabolic state of the regenerating bone defect. The increase in succinic acid, malic acid and several amino acids occurred together with reduced glutamine and reduced sugar-related metabolites, suggesting that the diabetic repair niche is not simply hyperglycemic but metabolically reorganized. Notably, valine, leucine and isoleucine were all higher in diabetic empty defects. This coordinated branched-chain amino acids (BCAA) shift supports a distinct amino acid imbalance within untreated diabetic repair tissue. Together, these findings indicate that untreated diabetic defects develop within a locally constrained metabolic environment characterized by altered energy-related metabolism and broad amino acid imbalance.

In plasma from empty-defect animals, the diabetic signature was weaker and less concordant with the local defect tissue. ROC analysis identified several nominal discriminatory metabolites, with methionine and glutaric acid remaining FDR-supported after Benjamini–Hochberg correction (Figure 4A; Supplementary Table S5). In the limma differential-abundance analysis, cholesterol and glycerol remained significant after correction, while additional nominal plasma differences highlighted broader diabetes-associated metabolic variation (Figure 4B; Supplementary Table S5). Together, these findings indicate that diabetes-associated plasma differences were detectable and biologically informative, although fewer metabolites met multiple-testing-corrected significance thresholds than in the regenerating defect tissue.

The plasma findings also differed qualitatively from the local defect profile. While several TCA-cycle-associated metabolites observed in the regenerating defect tissue, including citric acid, malic acid and succinic acid, were prominent local features, related plasma changes were primarily supported at the nominal level rather than as FDR-supported findings. Likewise, the local BCAA pattern was more evident within the defect tissue than in circulation, although nominal plasma differences suggested some systemic metabolic influence. This divergence indicates that diabetes imposed a stronger and more complex metabolic constraint within the regenerating bone defect than was detectable systemically. Thus, in the absence of scaffold treatment, the metabolic consequences of diabetes were most pronounced locally in the healing defect, while plasma profiling still captured complementary nominal metabolic alterations associated with the diabetic state.

#### Bone defect treated with SGBR

Within scaffold-treated defects, host-state differences remained prominent in the regenerating callus. ROC analysis identified 15 metabolites with nominal discriminatory power between diabetic and non-diabetic animals, of which 11 remained significant after Benjamini–Hochberg correction (Figure 3A; Supplementary Table S4). These corrected ROC findings were supported by limma differential-abundance analysis, which also identified adenosine and malic acid as FDR-significant differences (Figure 3B; Supplementary Table S4). Relative to scaffold-treated non-diabetic callus, diabetic callus showed higher isoleucine, malic acid, methionine, phenylalanine, threonine and succinic acid, together with lower glycerol-3-phosphate, maltose, sucrose, glutamine, arabitol and creatinine. Adenosine was also higher in diabetic scaffold-treated callus in the limma differential-abundance analysis. These findings show that scaffold implantation does not abolish the metabolic divergence between diabetic and non-diabetic healing. Substantial local differences persist despite scaffold treatment.

In plasma from scaffold-treated animals, the diabetic signature was again weaker than in the callus. ROC analysis identified 7 metabolites with nominal discriminatory power between diabetic and non-diabetic animals in scaffold-treated samples, but none remained significant after Benjamini–Hochberg correction (Figure 4A; Supplementary Table S6). In the limma differential-abundance analysis, 2-aminobutyric acid remained significant after correction and was lower in diabetic scaffold-treated animals (Figure 4B; Supplementary Table S6). The plasma analyses should be interpreted in light of the small sample size (*n* = 3–4). Plasma findings were therefore interpreted primarily through logFC, directionality and concordance with the local defect-tissue profile.

Overall, these data show that scaffold implantation is accompanied by broad metabolic changes in the regenerating callus, but that differences between diabetic and non-diabetic animals remain pronounced locally and only partially detectable in plasma. This indicates that the diabetic host continues to impose a local metabolic constraint even in the presence of the scaffold.

### SGBR promotes a regeneration-associated metabolic response in non-diabetic bone but only partial metabolic correction in diabetes

We next compared scaffold-treated defects with empty defects within each host condition to define the metabolic response associated with scaffold-guided bone regeneration itself (Figures 5 and 6). This analysis distinguishes host-state differences from scaffold-induced changes and asks whether scaffold implantation produces the same regenerative metabolic response in non-diabetic and diabetic animals. Metabolites were evaluated using ROC and limma differential-abundance analyses, as in the previous comparisons, and significance was assessed using Benjamini–Hochberg correction for multiple testing (Supplementary Tables S7–S10).

**Figure 5:**
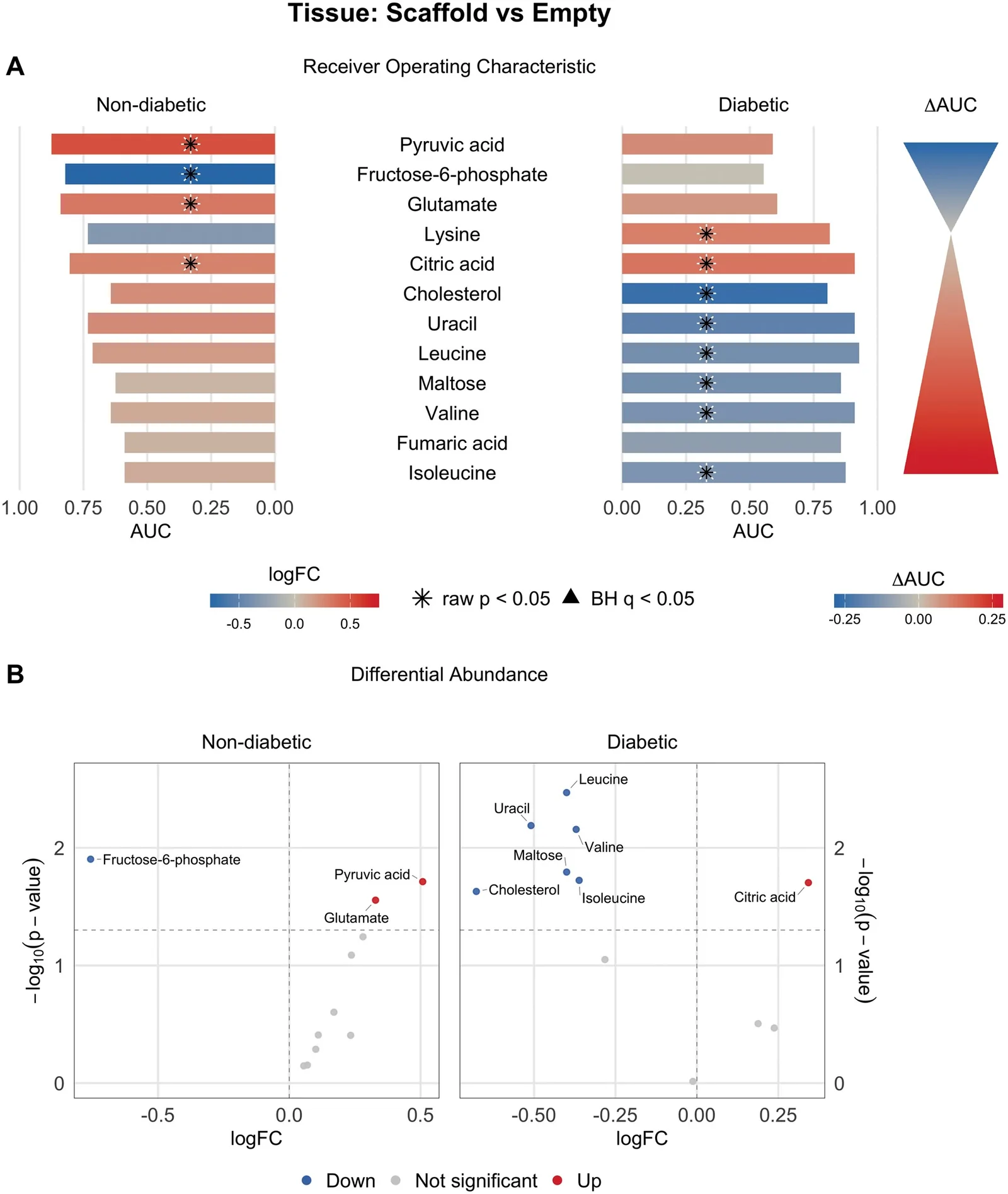
SGBR induces distinct metabolic responses in non-diabetic and diabetic regenerative defect tissue samples. (**A**) Horizontal bar plots show metabolites that discriminate between scaffold-treated and empty defects in non-diabetic animals (left, 7 scaffold samples and 8 empty samples) or diabetic animals (right, 7 scaffold samples and 8 empty samples). Bars represent AUC values, with colors indicating log fold change (logFC) relative to empty defects. Positive logFC (red) reflects increased metabolite abundance in scaffold-treated defects, while negative logFC (blue) indicates decreased abundance. Asterisks mark the host condition in which the metabolite met the predefined ROC display criteria of AUC ≥ 0.8 and raw *P* < 0.05 (unadjusted *P*-values from an unpaired, two-sided Student’s *t*-test). Metabolites are sorted by ΔAUC, the difference in AUC between diabetic and non-diabetic comparisons. No metabolite remained significant after Benjamini–Hochberg correction in the ROC analysis. (**B**) Volcano plots show limma differential-abundance analysis for the same comparisons. The *x*-axis represents logFC in scaffold-treated defects relative to empty defects, and the *y*-axis represents − log₁₀ *P*-values. Compounds are only included in the volcano plot if they reach an AUC ≥ 0.8 in (A). Red and blue labels indicate metabolites with nominal limma *P* < 0.05 and higher or lower abundance in scaffold-treated defects, respectively. No metabolite remained significant after Benjamini–Hochberg correction in the limma differential-abundance analysis.

**Figure 6:**
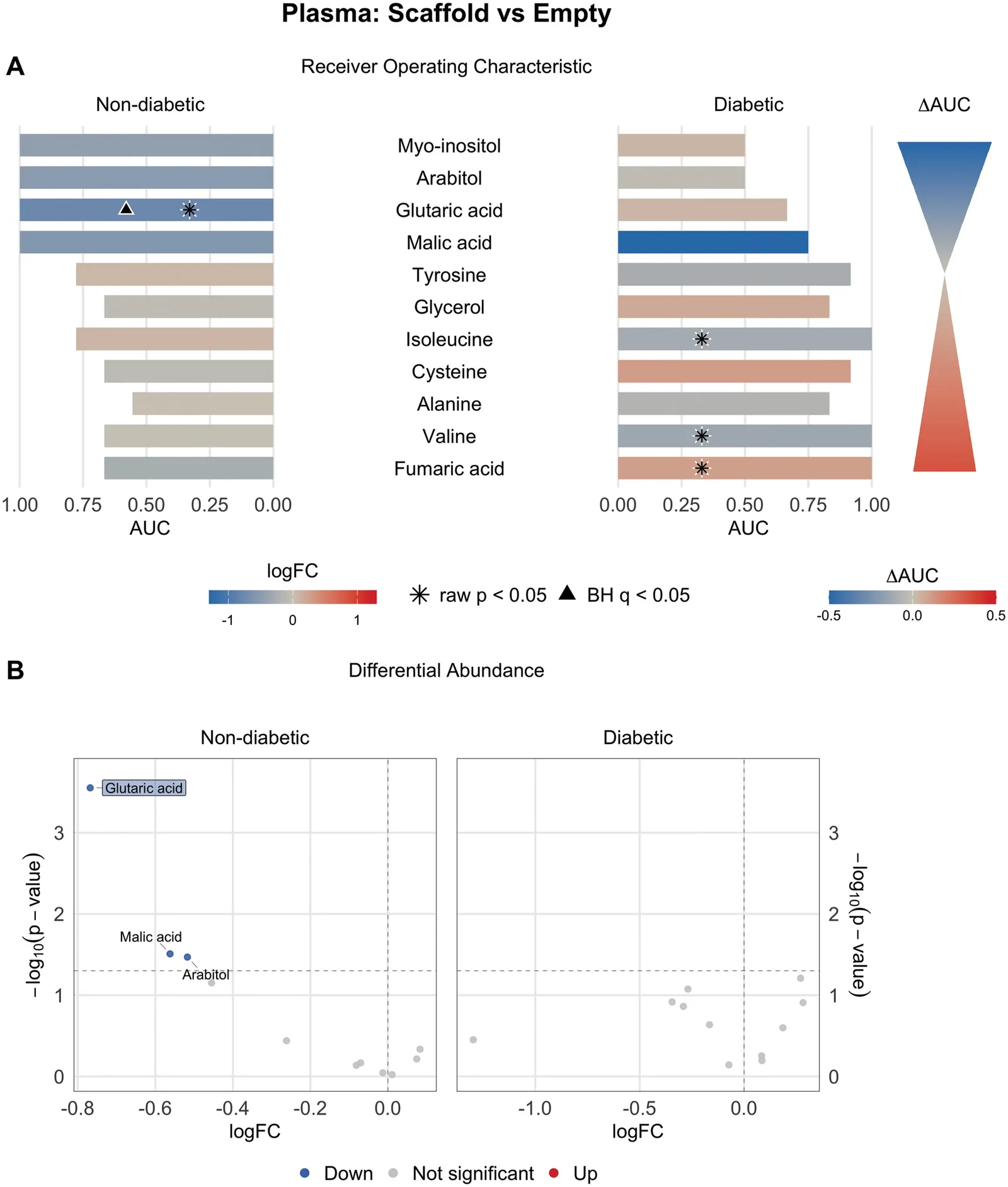
SGBR induces distinct plasma metabolic responses in non-diabetic and diabetic animals. (**A**) Horizontal bar plots show metabolites that discriminate between scaffold-treated and empty-defect animals in non-diabetic animals (left, 3 empty samples and 3 scaffold samples) or diabetic animals (right, 3 empty samples and 4 scaffold samples). Bars represent AUC values, with colors indicating log fold change (logFC) relative to empty-defect animals. Positive logFC (red) reflects increased metabolite abundance in scaffold-treated animals, while negative logFC (blue) indicates decreased abundance. Asterisks mark the host condition in which the metabolite met the predefined ROC display criteria of AUC ≥ 0.8 and raw *P* < 0.05 (unadjusted *P*-values from an unpaired, two-sided Student’s *t*-test). Metabolites are sorted by ΔAUC, the difference in AUC between diabetic and non-diabetic comparisons. (**B**) Volcano plots show limma differential-abundance analysis for the same comparisons. The *x*-axis represents logFC in scaffold-treated animals relative to empty-defect animals, and the *y*-axis represents − log₁₀ *P*-values. Compounds are only included in the volcano plot if they reach an AUC ≥ 0.8 in (A). Red and blue labels indicate metabolites with nominal limma *P* < 0.05 and higher or lower abundance in scaffold-treated animals, respectively. Glutaric acid was the only metabolite that remained significant after Benjamini–Hochberg correction (BH *q* < 0.05) in non-diabetic plasma and is highlighted by the black triangle and boxed label; no metabolite remained significant after correction in diabetic plasma.

#### Non-diabetic animals

In non-diabetic animals, scaffold treatment altered a small but coherent set of metabolites within the regenerating callus. ROC analysis identified four metabolites with nominal discriminatory power between scaffold-treated and empty defects, namely glutamate, fructose-6-phosphate, pyruvic acid and citric acid (Figure 5A; Supplementary Table S7). Three of these, fructose-6-phosphate, pyruvic acid and glutamate, were also nominally supported by limma differential-abundance analysis (Figure 5B; Supplementary Table S7). However, these local scaffold-associated metabolite differences did not remain significant after Benjamini–Hochberg correction. Relative to empty defects, scaffold-treated callus showed lower fructose-6-phosphate and higher citric acid, pyruvic acid and glutamate. These nominal changes are consistent with a local metabolic state characterized by greater substrate turnover and stronger engagement of pathways linked to energy generation and biosynthetic support.

This metabolic pattern aligned with the regenerative outcome. Scaffold implantation significantly improved bone regeneration in non-diabetic animals (Figure 2), and this improvement was accompanied by nominal local metabolic changes compatible with an active regenerative niche. Notably, glutamine abundance was elevated in the regenerating callus relative to sham bone in non-diabetic animals (Supplementary Figure S5), whereas glutamate was nominally increased in scaffold-treated compared with empty defects (Figure 5). Collectively, these findings indicate that in non-diabetic animals, successful scaffold-guided regeneration is associated with a regeneration-supportive local metabolic response involving coordinated changes in energy and amino acid metabolism. Together, these pathways may increase the availability of TCA cycle substrates, potentially supporting the synthesis of proline, ornithine and arginine, which are amino acids essential for collagen production and matrix formation. Their availability is consistent with the enhanced bone regeneration observed in scaffold-treated defects. This observation aligned with the established metabolic phenotype of osteoprogenitors and early osteoblast differentiation, which rely on glycolytic engagement to support rapid matrix deposition and mineralization [24–28].

By contrast, the plasma response in non-diabetic animals was limited but showed one robust scaffold-associated difference. ROC analysis identified glutaric acid as the only metabolite that remained significant after Benjamini–Hochberg correction in plasma from non-diabetic scaffold-treated versus empty-defect animals. Glutaric acid was also supported by limma differential-abundance analysis after correction (Figure 6A and B; Supplementary Table S9). Thus, in the non-diabetic setting, the scaffold-associated metabolic response was primarily local and not strongly reflected systemically. This finding supports the view that the metabolically relevant effects of scaffold implantation occur within the regenerating callus rather than in the circulation.

#### Diabetic animals

In diabetic animals, scaffold treatment also altered local metabolism, but the pattern differed from that observed in non-diabetic bone. ROC analysis identified eight metabolites with nominal discriminatory power between scaffold-treated and empty defects (Figure 5A; Supplementary Table S8). Seven of these were also nominally supported by limma differential-abundance analysis, namely leucine, uracil, valine, maltose, cholesterol, citric acid and isoleucine (Figure 5B; Supplementary Table S8). However, none of the local scaffold-associated differences in diabetic defects remained significant after Benjamini–Hochberg correction in either ROC or limma differential-abundance analysis. SGBR treatment increased citric acid and lysine levels, while cholesterol, isoleucine, leucine, maltose, uracil and valine were reduced compared with empty defects. The nominal reduction in valine, leucine and isoleucine is notable because it indicates partial normalization of the elevated BCAA state observed in diabetic empty defects (Figure 3A). However, this partial correction was not accompanied by restoration of glutamine–glutamate-related metabolism or by improved bone formation.

In the plasma samples, the diabetic scaffold response was again limited and statistical interpretation constrained due to sample size (*n* = 3–4). ROC analysis identified three nominal discriminatory metabolites, but none remained significant after Benjamini–Hochberg correction, and none was supported by limma differential-abundance analysis after correction (Figure 6A and B; Supplementary Table S10). Thus, scaffold treatment in diabetic animals induced a narrower and less coherent metabolic response than in non-diabetic animals.

This contrast has two implications. First, the scaffold did not simply fail because it was structurally inadequate, since the same construct promoted regeneration in non-diabetic bone. Second, the diabetic host retained the capacity for some metabolic response to scaffold implantation, but this response was incomplete and did not reproduce the metabolic state associated with effective regeneration. In that sense, diabetic defects did not show a complete absence of scaffold responsiveness. Rather, they showed a narrowed scaffold-associated metabolic response that was insufficient to translate into regenerative benefit.

The BCAA pattern helps clarify this distinction. BCAAs are essential amino acids, i.e., valine, leucine and isoleucine, that bypass hepatic metabolism and are rapidly taken up and oxidized by peripheral tissues, serving as metabolic fuels and regulators of energy balance during exercise and severe illness [29–31]. In intact bone, BCAA levels did not differ between diabetic and non-diabetic animals, whereas valine and isoleucine were elevated in diabetic plasma (Figure 1). During defect healing, diabetic empty defects accumulated BCAAs within the callus, and this accumulation coincided with broader amino acid dysregulation (Supplementary Figure S5). Previous *in vivo* studies have shown that high BCAA concentrations impair bone microarchitecture and disrupt energy metabolism [32]. *In vitro*, BCAAs have been shown to inhibit MC3T3E1 pre-osteoblast proliferation, osteogenic differentiation and glycolysis, likely via HIF1α and GLUT1 regulation [32], while also promoting osteoclast maturation [33]. In the present study, scaffold treatment was associated with reduced BCAA abundance in diabetic callus, but bone regeneration remained poor. This argues that partial normalization of BCAA abundance alone is not sufficient to restore scaffold-guided regeneration under diabetic conditions.

Taken together, these data indicate that SGBR is associated with distinct and condition-dependent metabolic shifts. In non-diabetic bone, scaffold treatment was associated with a regeneration-associated metabolic response marked by nominal local changes in energy and amino acid metabolism, together with improved bone regeneration. In diabetic animals, this response was narrower, did not remain robust after multiple-testing correction at the local metabolite level, and was less evident in plasma. This pattern is consistent with the lack of scaffold-associated improvement in diabetic bone regeneration.

### Glutamate-related metabolism as a candidate translational axis in diabetic scaffold-guided regeneration

Within the metabolic patterns described above, glutamate-related metabolism emerged as the most biologically coherent axis linking host state, scaffold response and regenerative outcome. Glutamate occupies a central position at the interface of bioenergetics, biosynthesis and redox balance (Figure 7) [34–37]. It is primarily synthesized from glutamine via glutaminase, and can also be generated through the transamination of α-ketoglutarate (α-KG), a key intermediate of the TCA cycle (Figure 7). A functional role for glutamate-related pathways in bone has also been described, including effects on bone homeostasis, matrix production and repair [34, 35, 38–40]. This broader framework provides a plausible context for interpreting the present dataset.

**Figure 7:**
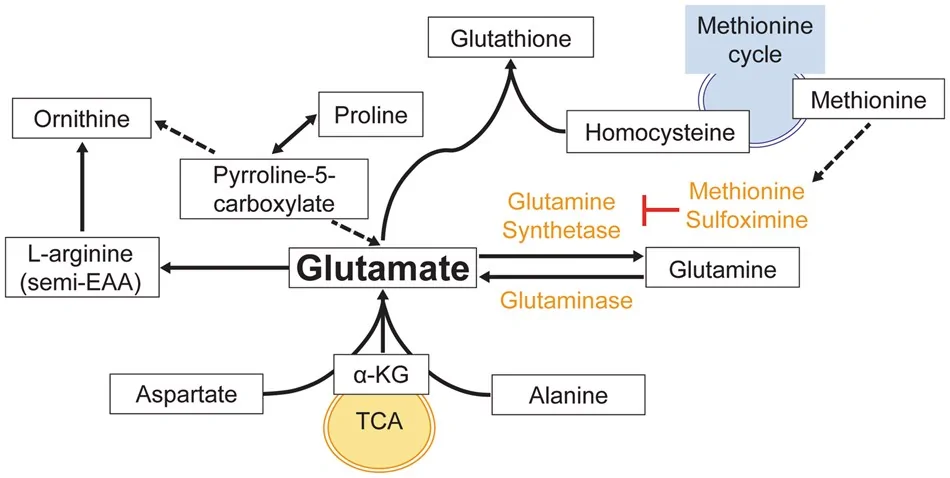
Glutamate is depicted at the center of multiple intersecting pathways that support energy production, biosynthesis and redox homeostasis. It is reversibly converted to α-ketoglutarate (α-KG) to fuel the TCA cycle for ATP generation. Through pyrroline-5-carboxylate, glutamate donates carbon and nitrogen for proline synthesis, a key collagen precursor, and via ornithine it feeds into the arginine pathway. Transamination also yields aspartate and alanine, linking to urea cycling and gluconeogenesis. Glutamine synthetase incorporates ammonia into glutamate to form glutamine, which is a reaction that is specifically inhibited by methionine sulfoximine, while glutaminase returns glutamine to glutamate. Glutamate is further tied to the methionine cycle: homocysteine is methylated to regenerate methionine, and S-adenosylmethionine-derived methyl groups support methylation reactions. Finally, glutamate supplies the γ-glutamyl moiety of glutathione, underpinning antioxidant defenses against oxidative stress.

In this study, lower basal glutamate abundance was observed in diabetic bone relative to non-diabetic bone (Figure 1B). During defect healing, diabetic animals also showed lower glutamine levels in the regenerating callus than non-diabetic animals, whereas glutamate itself was not significantly changed in that comparison (Figure 3 and Supplementary Figure S5). On the other hand, in non-diabetic rats, which demonstrated the most robust bone regeneration response following PCL scaffold treatment, glutamine was elevated in regenerating tissue relative to sham bone, and glutamate showed a positive trend toward higher abundance in scaffold-treated compared with empty defects. Although this scaffold-associated glutamate difference did not remain significant after correction for multiple testing (Figure 5), the overall pattern is encouraging and aligns with enhanced regenerative activity. Taken together, these findings highlight glutamate-related metabolism as a promising pathway associated with successful scaffold-guided regeneration and provide a strong rationale for future studies to further validate and refine the metabolite-level scaffold response.

This interpretation is also compatible with prior evidence. *In vitro*, α-KG supplementation enhanced the osteogenic capacity of mesenchymal stem cells (MSCs) from diabetic donors [9]. Glutamine’s positive role in bone repair has also been well documented [41–43], although elevated systemic levels of glutamate have been implicated in diabetes pathogenesis [44, 45]. In the exploratory clinical dataset, plasma α-KG increased in non-diabetic fracture patients between 0.5 days and 3 months post-operatively (Supplementary Figure S6). This temporal increase follows the same direction as the glutamate-related metabolic features observed during effective healing in non-diabetic rats, supporting the biological relevance of glutamate/α-KG-related metabolism during bone repair across species. The single available T2D sample at 3 months did not show a comparable α-KG increase, which is qualitatively consistent with the attenuated glutamate-related response observed in ZDF rats. However, because only one T2D patient was available at the 3-month follow-up, this qualitative absence of α-KG upregulation remains descriptive and hypothesis-generating. Together, the preclinical data and exploratory clinical observation support further investigation of glutamate-related metabolism as a candidate axis in bone repair and scaffold-guided regeneration.

The significance of glutamate-related metabolism is that it identifies a metabolically relevant dimension of scaffold performance that is consistently weakened in diabetes. This has practical implications for biomaterials research. Scaffold design is often optimized for architecture, mechanics or bioactivity. The present findings suggest that compatibility with the host metabolic environment may represent an additional determinant of success in compromised healing settings such as diabetes.

The present study establishes a 6-week endpoint framework linking host state, scaffold-associated metabolic profiles and regenerative outcome. This framework provides a basis for moving from association to mechanism. Future studies should define when glutamate-, α-KG- and BCAA-related changes emerge during healing and validate these nodes using targeted quantitative assays. Because the present GC-MS panel focused on polar metabolites, broader metabolite profiling, including lipidomic approaches, could further determine whether additional metabolite classes contribute to impaired scaffold-guided regeneration in diabetes. Local supplementation or scaffold-based delivery of glutamate- or α-KG-related metabolites would then provide a direct test of whether restoring this candidate metabolic axis can improve regeneration in diabetic bone defects. Together, these studies would help delineate causal mechanisms and guide metabolically adapted therapeutic strategies for T2D-associated bone-regeneration failure.

## Conclusion

By integrating plasma and regenerating callus metabolomics across non-diabetic and diabetic ZDF rats, this study identifies metabolic constraints underlying impaired bone regeneration in diabetes. Diabetic animals exhibited a systemic metabolic signature marked by elevated sugar-derived substrates and disrupted amino acid homeostasis. These alterations were reflected locally in empty bone defects and were associated with limited regenerative progression. Within this constrained environment, PCL scaffold implantation selectively altered local amino acid-related metabolic profiles. In non-diabetic animals, scaffold implantation improved bone regeneration and was accompanied by nominal local changes in glutamate and energy-related metabolites, while glutamine was elevated in regenerating tissue relative to sham bone. By contrast, in diabetic animals, scaffold implantation was associated with reduced levels of BCAAs without restoration of glutamate-related metabolism, indicating an incomplete metabolic correction. The persistent failure to restore glutamate-related metabolism paralleled the limited ability of PCL scaffolds to rescue bone regeneration under diabetic conditions. Overall, the data suggest that PCL scaffold implantation reshapes the local metabolome toward a regeneration-supportive state, but that this response depends on host metabolic state. These findings highlight host metabolic state as an important consideration for biomaterial strategies aimed at effective scaffold-guided bone regeneration in metabolically compromised settings, such as diabetes.

## Supplementary Material

rbag177_Supplementary_Data
