# Supplementary material for: Polycaprolactone scaffolds promote a regeneration-supportive metabolic response attenuated in diabetes
Source: Regen Biomater. 2026 Aug 17;13:rbag177. doi: 10.1093/rb/rbag177 (PMC13644532; doi:10.1093/rb/rbag177)
Supplement: Supplementary file 1 [file rbag177_supplementary_data.zip › Supplementary_Data/Supplementary_Tables.docx]

**Supplementary Table S1**. **Complete metabolite-level statistics for the femoral sham baseline comparison between diabetic and non-diabetic animals.** AUC values indicate univariate discriminatory performance. log₂FC was calculated as diabetic relative to non-diabetic sham animals. p-values were calculated using unpaired two-sided Student’s t-tests. Benjamini–Hochberg q-values were calculated across all tested metabolites in this compartment-specific comparison family. “Direction” indicates whether each metabolite was increased or decreased in diabetic samples. The comparison included non-diabetic sham n = 5 and diabetic sham n = 4.

| **Metabolite** | **AUC** | **log_2_FC** | **p-value** | **BH q-value** | **Direction** |
| --- | --- | --- | --- | --- | --- |
| Glucose | 1.0000 | 1.8853 | 0.0007 | 0.0294 | Up-regulated |
| Glucuronic acid | 1.0000 | 2.4683 | 0.0026 | 0.0424 | Up-regulated |
| Sucrose | 1.0000 | 1.8512 | 0.0041 | 0.0424 | Up-regulated |
| Maltose | 1.0000 | 1.5233 | 0.0042 | 0.0424 | Up-regulated |
| Lysine | 1.0000 | 2.0347 | 0.0128 | 0.1027 | Up-regulated |
| β-alanine | 0.8000 | -0.8893 | 0.0264 | 0.1753 | Down-regulated |
| Glutamate | 0.9500 | -0.5995 | 0.0347 | 0.1753 | Down-regulated |
| Serine | 0.9500 | -0.6890 | 0.0407 | 0.1753 | Down-regulated |
| Phenylalanine | 0.9000 | -0.9646 | 0.0462 | 0.1753 | Down-regulated |
| Threonine | 0.9500 | -0.7528 | 0.0467 | 0.1753 | Down-regulated |
| Glyceric acid | 0.9000 | -0.9526 | 0.0502 | 0.1753 | Down-regulated |
| Methionine | 0.9500 | -1.3442 | 0.0526 | 0.1753 | Down-regulated |
| Malic acid | 0.8000 | -0.6718 | 0.0604 | 0.1857 | Down-regulated |
| Fructose | 0.8000 | -2.0003 | 0.0773 | 0.2207 | Down-regulated |
| Arabitol | 0.8500 | 0.9493 | 0.1424 | 0.3796 | Up-regulated |
| Isoleucine | 0.8000 | -0.5874 | 0.1542 | 0.3856 | Down-regulated |
| Alanine | 0.7500 | 0.2961 | 0.1837 | 0.4323 | Up-regulated |
| 4-hydroxyproline | 0.7500 | -0.4921 | 0.2027 | 0.4504 | Down-regulated |
| Fumaric acid | 0.7500 | -0.3352 | 0.2286 | 0.4813 | Down-regulated |
| Uracil | 0.8000 | -0.5544 | 0.2807 | 0.5556 | Down-regulated |
| Glutamine | 0.6000 | -0.5969 | 0.2917 | 0.5556 | Down-regulated |
| Urea | 0.7000 | -0.2944 | 0.3638 | 0.6615 | Down-regulated |
| Succinic acid | 0.7500 | -0.6624 | 0.4108 | 0.7085 | Down-regulated |
| Cholesterol | 0.6000 | 0.3329 | 0.4413 | 0.7085 | Up-regulated |
| Glycerol-3-phosphate | 0.6500 | 0.2515 | 0.4439 | 0.7085 | Up-regulated |
| Uric acid | 0.5500 | -0.3266 | 0.4861 | 0.7085 | Down-regulated |
| Citric acid | 0.6000 | 0.2339 | 0.4874 | 0.7085 | Up-regulated |
| Pyruvic acid | 0.7000 | 0.2793 | 0.4960 | 0.7085 | Up-regulated |
| Asparagine | 0.6500 | 0.2084 | 0.5366 | 0.7200 | Up-regulated |
| Tyrosine | 0.6500 | -0.4767 | 0.5442 | 0.7200 | Down-regulated |
| Glycerol | 0.6000 | -0.2809 | 0.5580 | 0.7200 | Down-regulated |
| Adenosine | 0.6000 | -0.4906 | 0.6226 | 0.7782 | Down-regulated |
| Tryptophan | 0.6500 | -0.0727 | 0.6725 | 0.8151 | Down-regulated |
| Fructose-6-phosphate | 0.5500 | -0.1723 | 0.7363 | 0.8663 | Down-regulated |
| Creatinine | 0.6000 | 0.1720 | 0.8002 | 0.9031 | Up-regulated |
| Lactic acid | 0.5500 | 0.0683 | 0.8147 | 0.9031 | Up-regulated |
| Uridine | 0.6000 | 0.0602 | 0.8408 | 0.9031 | Up-regulated |
| Valine | 0.6000 | -0.0525 | 0.8579 | 0.9031 | Down-regulated |
| Hypotaurine | 0.5000 | 0.0135 | 0.9597 | 0.9843 | Up-regulated |
| Leucine | 0.6500 | 0.0076 | 0.9861 | 0.9861 | Up-regulated |

**Supplementary Table S2**. **Complete metabolite-level statistics for the plasma sham baseline comparison between diabetic and non-diabetic animals.** AUC values indicate univariate discriminatory performance. log₂FC was calculated as diabetic relative to non-diabetic sham animals. p-values were calculated using unpaired two-sided Student’s t-tests. Benjamini–Hochberg q-values were calculated across all tested metabolites in this compartment-specific comparison family. “Direction” indicates whether each metabolite was increased or decreased in diabetic samples. The comparison included non-diabetic sham n = 3 and diabetic sham n = 4.

| **Metabolite** | **AUC** | **log_2_FC** | **p-value** | **BH q-value** | **Direction** |
| --- | --- | --- | --- | --- | --- |
| Glucose | 1.0000 | 1.1876 | 0.0005 | 0.0165 | Up-regulated |
| Tryptophan | 1.0000 | -0.8308 | 0.0047 | 0.0656 | Down-regulated |
| Valine | 1.0000 | 1.1334 | 0.0060 | 0.0656 | Up-regulated |
| Isoleucine | 1.0000 | 1.0839 | 0.0160 | 0.1148 | Up-regulated |
| Cholesterol | 1.0000 | 1.3357 | 0.0174 | 0.1148 | Up-regulated |
| Myo-inositol | 1.0000 | 0.4235 | 0.0209 | 0.1148 | Up-regulated |
| Serine | 1.0000 | -0.5184 | 0.0283 | 0.1228 | Down-regulated |
| Fumaric acid | 1.0000 | 0.6403 | 0.0318 | 0.1228 | Up-regulated |
| Maltose | 1.0000 | 1.3248 | 0.0361 | 0.1228 | Up-regulated |
| 2-aminobutyric acid | 1.0000 | 0.7103 | 0.0372 | 0.1228 | Up-regulated |
| Citric acid | 1.0000 | 0.2850 | 0.0432 | 0.1295 | Up-regulated |
| Leucine | 0.9167 | 0.5360 | 0.0548 | 0.1507 | Up-regulated |
| Uracil | 0.8333 | -0.6438 | 0.0683 | 0.1688 | Down-regulated |
| Pyruvic acid | 0.8333 | 0.6354 | 0.0716 | 0.1688 | Up-regulated |
| Sucrose | 0.7500 | 0.4504 | 0.1737 | 0.3821 | Up-regulated |
| Urea | 0.8333 | 0.2423 | 0.1948 | 0.4017 | Up-regulated |
| Lactic acid | 0.8333 | 0.4580 | 0.2139 | 0.4153 | Up-regulated |
| Succinic acid | 0.8333 | -0.2315 | 0.2820 | 0.5169 | Down-regulated |
| Glutamine | 0.6667 | -0.4724 | 0.3461 | 0.5955 | Down-regulated |
| Glyceric acid | 0.7500 | 0.5758 | 0.3796 | 0.5955 | Up-regulated |
| Tyrosine | 0.5833 | -0.1462 | 0.4142 | 0.5955 | Down-regulated |
| Arabitol | 0.7500 | -0.2291 | 0.4291 | 0.5955 | Down-regulated |
| Glutaric acid | 0.5833 | 0.3423 | 0.4316 | 0.5955 | Up-regulated |
| Threonine | 0.6667 | -0.1437 | 0.4498 | 0.5955 | Down-regulated |
| Alanine | 0.5833 | 0.2511 | 0.4511 | 0.5955 | Up-regulated |
| Malic acid | 0.6667 | 0.3219 | 0.4747 | 0.6025 | Up-regulated |
| Glycerol | 0.5833 | 0.1803 | 0.5314 | 0.6495 | Up-regulated |
| Proline | 0.6667 | 0.2093 | 0.5552 | 0.6543 | Up-regulated |
| 4-hydroxyproline | 0.6667 | -0.2464 | 0.5851 | 0.6632 | Down-regulated |
| Uric acid | 0.5000 | 0.1407 | 0.6029 | 0.6632 | Up-regulated |
| Glutamate | 0.6667 | -0.0833 | 0.7746 | 0.8246 | Down-regulated |
| Cysteine | 0.5833 | -0.0350 | 0.9031 | 0.9313 | Down-regulated |
| Methionine | 0.5833 | -0.0015 | 0.9922 | 0.9922 | Down-regulated |

| **Metabolite** | **AUC** | **logFC** | **ROC p** | **ROC q** | **DE p** | **DE q** |
| --- | --- | --- | --- | --- | --- | --- |
| Succinic acid | 1.0000 | 1.6599 | 0.0000 | 0.0000 | 0.0000 | 0.0000 |
| Uracil | 1.0000 | 0.7669 | 0.0000 | 0.0001 | 0.0000 | 0.0001 |
| Glycerol-3-phosphate | 1.0000 | -1.0677 | 0.0000 | 0.0001 | 0.0000 | 0.0000 |
| Creatinine | 1.0000 | -1.2169 | 0.0000 | 0.0004 | 0.0000 | 0.0000 |
| Glutamine | 0.9844 | -0.9830 | 0.0001 | 0.0005 | 0.0000 | 0.0001 |
| Isoleucine | 0.9531 | 0.8868 | 0.0001 | 0.0005 | 0.0000 | 0.0001 |
| Threonine | 0.9531 | 0.6888 | 0.0001 | 0.0005 | 0.0001 | 0.0003 |
| β-alanine | 0.9531 | 0.7879 | 0.0001 | 0.0007 | 0.0001 | 0.0005 |
| Methionine | 0.9375 | 1.2601 | 0.0003 | 0.0013 | 0.0000 | 0.0002 |
| Valine | 0.9375 | 0.5044 | 0.0005 | 0.0019 | 0.0007 | 0.0019 |
| Glucuronic acid | 0.9531 | -0.7160 | 0.0005 | 0.0019 | 0.0002 | 0.0009 |
| Arabitol | 0.9531 | -0.7710 | 0.0015 | 0.0046 | 0.0004 | 0.0015 |
| Serine | 0.9063 | 0.6754 | 0.0016 | 0.0046 | 0.0007 | 0.0019 |
| Phenylalanine | 0.9063 | 0.6591 | 0.0018 | 0.0046 | 0.0008 | 0.0019 |
| Malic acid | 0.8906 | 0.6922 | 0.0018 | 0.0046 | 0.0007 | 0.0019 |
| Leucine | 0.8438 | 0.4530 | 0.0039 | 0.0093 | 0.0045 | 0.0081 |
| Maltose | 0.8750 | -0.6027 | 0.0041 | 0.0093 | 0.0007 | 0.0019 |
| Tyrosine | 0.8906 | -0.6454 | 0.0045 | 0.0094 | 0.0022 | 0.0046 |
| Sucrose | 0.8906 | -1.1733 | 0.0046 | 0.0094 | 0.0022 | 0.0046 |
| Citric acid | 0.8906 | -0.3171 | 0.0057 | 0.0110 | 0.0173 | 0.0285 |
| Glyceric acid | 0.8750 | 0.7005 | 0.0059 | 0.0110 | 0.0033 | 0.0063 |
| Urea | 0.8438 | 0.6384 | 0.0071 | 0.0126 | 0.0032 | 0.0063 |
| Fructose-6-phosphate | 0.7969 | -0.9119 | 0.0316 | 0.0535 | 0.0120 | 0.0207 |
| Alanine | 0.7500 | 0.3278 | 0.0329 | 0.0535 | 0.0336 | 0.0533 |
| Fumaric acid | 0.7656 | 0.3928 | 0.0500 | 0.0781 | 0.0361 | 0.0548 |
| Tryptophan | 0.7188 | -0.2429 | 0.0634 | 0.0952 | 0.0800 | 0.1086 |
| Fructose | 0.6875 | NA | 0.0776 | 0.1121 | NA | NA |
| Glucose | 0.7500 | -0.2273 | 0.0922 | 0.1284 | 0.1083 | 0.1372 |
| Cholesterol | 0.7188 | 0.4397 | 0.0997 | 0.1341 | 0.0874 | 0.1145 |
| Adenosine | 0.7344 | 0.8049 | 0.1178 | 0.1532 | 0.0644 | 0.0906 |
| Glutamate | 0.7500 | 0.1971 | 0.1476 | 0.1857 | 0.1649 | 0.2021 |
| Asparagine | 0.6719 | -0.3064 | 0.1730 | 0.2109 | 0.0625 | 0.0906 |
| Glycerol | 0.6719 | 0.2020 | 0.3182 | 0.3760 | 0.4141 | 0.4918 |
| 4-hydroxyproline | 0.6094 | 0.0811 | 0.4740 | 0.5385 | 0.5295 | 0.5918 |
| Uridine | 0.6250 | -0.1576 | 0.4833 | 0.5385 | 0.4439 | 0.5111 |
| Lactic acid | 0.5625 | 0.0550 | 0.6620 | 0.7172 | 0.6867 | 0.7278 |
| Pyruvic acid | 0.5156 | -0.1047 | 0.7262 | 0.7478 | 0.6895 | 0.7278 |
| Hypotaurine | 0.5313 | -0.0378 | 0.7287 | 0.7478 | 0.7622 | 0.7828 |
| Uric acid | 0.6094 | -0.0414 | 0.8676 | 0.8676 | 0.8540 | 0.8540 |

**Supplementary Table S3. Complete metabolite-level statistics for the diabetic versus non-diabetic comparison in empty regenerating defects.** AUC values indicate ROC-based discriminatory performance. logFC was calculated as diabetic relative to non-diabetic animals; positive values indicate higher abundance in diabetic animals and negative values lower abundance. ROC p and ROC q indicate the raw and Benjamini–Hochberg-adjusted p-values from the ROC-associated unpaired two-sided Student’s t-test. DE p and DE q indicate the raw and Benjamini–Hochberg-adjusted p-values from the limma differential-abundance analysis. NA indicates insufficient GC–MS detection for estimation of the corresponding statistic. The comparison included non-diabetic empty-defect n = 8 and diabetic empty-defect n = 8.

| **Metabolite** | **AUC** | **logFC** | **ROC p** | **ROC q** | **DE p** | **DE q** |
| --- | --- | --- | --- | --- | --- | --- |
| Sucrose | 1.0000 | -1.4583 | <0.0001 | 0.0002 | <0.0001 | <0.0001 |
| Maltose | 1.0000 | -1.0578 | <0.0001 | 0.0003 | <0.0001 | <0.0001 |
| Phenylalanine | 1.0000 | 0.6671 | <0.0001 | 0.0003 | <0.0001 | 0.0003 |
| Succinic acid | 0.9796 | 1.5302 | 0.0005 | 0.0047 | <0.0001 | 0.0004 |
| Creatinine | 0.9796 | -0.8320 | 0.0006 | 0.0047 | 0.0002 | 0.0017 |
| Methionine | 0.8980 | 1.0542 | 0.0017 | 0.0112 | 0.0004 | 0.0024 |
| Isoleucine | 0.8980 | 0.4243 | 0.0024 | 0.0140 | 0.0031 | 0.0161 |
| Threonine | 0.9388 | 0.3356 | 0.0029 | 0.0147 | 0.0080 | 0.0254 |
| Glutamine | 0.9592 | -0.6944 | 0.0069 | 0.0305 | 0.0042 | 0.0176 |
| Arabitol | 0.9184 | -0.6004 | 0.0100 | 0.0398 | 0.0034 | 0.0161 |
| Glycerol-3-phosphate | 0.8776 | -0.5284 | 0.0123 | 0.0446 | 0.0066 | 0.0227 |
| Adenosine | 0.8776 | 1.3682 | 0.0185 | 0.0602 | 0.0048 | 0.0181 |
| Tryptophan | 0.8571 | -0.2472 | 0.0196 | 0.0602 | 0.0465 | 0.1018 |
| Malic acid | 0.8571 | 0.4582 | 0.0220 | 0.0629 | 0.0111 | 0.0324 |
| β-alanine | 0.8776 | 0.4096 | 0.0257 | 0.0686 | 0.0327 | 0.0829 |
| Pyruvic acid | 0.7755 | -0.3752 | 0.0495 | 0.1236 | 0.0422 | 0.1003 |
| Fructose | 0.7857 | NA | 0.0602 | 0.1417 | NA | NA |
| Serine | 0.6531 | 0.1766 | 0.0642 | 0.1427 | 0.1655 | 0.2515 |
| Urea | 0.7551 | 0.2908 | 0.0731 | 0.1539 | 0.0785 | 0.1360 |
| Asparagine | 0.7347 | -0.3785 | 0.0844 | 0.1689 | 0.0188 | 0.0510 |
| Citric acid | 0.8163 | -0.2554 | 0.0894 | 0.1702 | 0.0787 | 0.1360 |
| Uridine | 0.7347 | -0.3723 | 0.0953 | 0.1732 | 0.0652 | 0.1239 |
| Glyceric acid | 0.7347 | 0.5673 | 0.1095 | 0.1904 | 0.0617 | 0.1235 |
| Glucuronic acid | 0.6735 | -0.4013 | 0.1154 | 0.1904 | 0.0482 | 0.1018 |
| Uric acid | 0.7347 | -0.3567 | 0.1190 | 0.1904 | 0.0916 | 0.1513 |
| Cholesterol | 0.7143 | -0.4712 | 0.1449 | 0.2229 | 0.1579 | 0.2500 |
| Lysine | 0.6327 | NA | 0.2324 | 0.3443 | NA | NA |
| Tyrosine | 0.5510 | -0.2348 | 0.2682 | 0.3831 | 0.2445 | 0.3573 |
| Leucine | 0.6735 | -0.1170 | 0.2792 | 0.3852 | 0.3686 | 0.5002 |
| 4-hydroxyproline | 0.5510 | 0.1096 | 0.3160 | 0.4213 | 0.3572 | 0.5002 |
| Fructose-6-phosphate | 0.6327 | -0.1675 | 0.4177 | 0.5389 | 0.3894 | 0.5103 |
| Lactic acid | 0.5918 | -0.1170 | 0.5637 | 0.7047 | 0.5249 | 0.6649 |
| Alanine | 0.5306 | -0.0414 | 0.6949 | 0.8424 | 0.7231 | 0.8864 |
| Glutamate | 0.5306 | 0.0566 | 0.7842 | 0.8895 | 0.7649 | 0.8931 |
| Fumaric acid | 0.5102 | 0.0418 | 0.8013 | 0.8895 | 0.7921 | 0.8931 |
| Hypotaurine | 0.5714 | 0.0402 | 0.8124 | 0.8895 | 0.8087 | 0.8931 |
| Valine | 0.5306 | 0.0232 | 0.8228 | 0.8895 | 0.8461 | 0.8931 |
| Glycerol | 0.5102 | -0.0499 | 0.8587 | 0.9039 | 0.8377 | 0.8931 |
| Uracil | 0.5102 | 0.0211 | 0.8934 | 0.9163 | 0.9009 | 0.9252 |
| Glucose | 0.6531 | -0.0006 | 0.9983 | 0.9983 | 0.9980 | 0.9980 |

**Supplementary Table S4. Complete metabolite-level statistics for the diabetic versus non-diabetic comparison in scaffold-treated regenerating defects.** AUC values indicate ROC-based discriminatory performance. logFC was calculated as diabetic relative to non-diabetic animals; positive values indicate higher abundance in diabetic animals and negative values lower abundance. ROC p and ROC q indicate the raw and Benjamini–Hochberg-adjusted p-values from the ROC-associated unpaired two-sided Student’s t-test. DE p and DE q indicate the raw and Benjamini–Hochberg-adjusted p-values from the limma differential-abundance analysis. NA indicates insufficient GC–MS detection for estimation of the corresponding statistic. The comparison included non-diabetic scaffold-treated n = 7 and diabetic scaffold-treated n = 7.

| **Metabolite** | **AUC** | **logFC** | **ROC p** | **ROC q** | **DE p** | **DE q** |
| --- | --- | --- | --- | --- | --- | --- |
| Methionine | 1.0000 | -0.2216 | 0.0004 | 0.0119 | 0.0077 | 0.0505 |
| Glutaric acid | 1.0000 | -0.5101 | 0.0023 | 0.0383 | 0.0075 | 0.0505 |
| Cholesterol | 1.0000 | -0.6441 | 0.0056 | 0.0621 | 0.0020 | 0.0433 |
| Glycerol | 1.0000 | -0.4020 | 0.0080 | 0.0661 | 0.0026 | 0.0433 |
| 2-aminobutyric acid | 1.0000 | -0.7184 | 0.0113 | 0.0743 | 0.0066 | 0.0505 |
| Serine | 1.0000 | -0.2402 | 0.0160 | 0.0882 | 0.0191 | 0.0789 |
| Threonine | 1.0000 | -0.2674 | 0.0220 | 0.1037 | 0.0191 | 0.0789 |
| Tryptophan | 1.0000 | 0.5791 | 0.0298 | 0.1231 | 0.0163 | 0.0789 |
| Isoleucine | 1.0000 | -0.1494 | 0.0385 | 0.1411 | 0.0832 | 0.2287 |
| Fumaric acid | 1.0000 | -0.5370 | 0.0643 | 0.1953 | 0.0443 | 0.1461 |
| Uracil | 1.0000 | 0.4999 | 0.0651 | 0.1953 | 0.0489 | 0.1468 |
| Alanine | 1.0000 | 0.3514 | 0.0800 | 0.2199 | 0.0372 | 0.1365 |
| Cysteine | 1.0000 | -0.3472 | 0.1176 | 0.2985 | 0.1032 | 0.2447 |
| Urea | 0.8889 | -0.1505 | 0.1336 | 0.3148 | 0.1089 | 0.2447 |
| Glucose | 0.7778 | -0.1829 | 0.1530 | 0.3347 | 0.1112 | 0.2447 |
| Leucine | 0.6667 | 0.7547 | 0.1623 | 0.3347 | 0.1971 | 0.2920 |
| Pyruvic acid | 0.6667 | -0.2003 | 0.1853 | 0.3432 | 0.1402 | 0.2731 |
| Arabitol | 0.8889 | -0.3108 | 0.1918 | 0.3432 | 0.1520 | 0.2787 |
| Uric acid | 0.7778 | 0.3871 | 0.2228 | 0.3432 | 0.1708 | 0.2819 |
| Valine | 0.7778 | -0.0597 | 0.2230 | 0.3432 | 0.3761 | 0.4460 |
| Sucrose | 0.7778 | -0.6999 | 0.2324 | 0.3432 | 0.1407 | 0.2731 |
| Tyrosine | 0.8889 | 0.2032 | 0.2385 | 0.3432 | 0.2837 | 0.3901 |
| Glutamate | 0.7778 | 0.4808 | 0.2392 | 0.3432 | 0.1651 | 0.2819 |
| Myo-inositol | 0.6667 | -0.3319 | 0.2993 | 0.4116 | 0.2032 | 0.2920 |
| Glutamine | 0.8889 | 0.4879 | 0.3158 | 0.4168 | 0.2035 | 0.2920 |
| Malic acid | 0.6667 | -0.1993 | 0.4347 | 0.5216 | 0.3784 | 0.4460 |
| Citric acid | 0.7778 | -0.3086 | 0.4416 | 0.5216 | 0.3555 | 0.4460 |
| Maltose | 0.5556 | -0.4743 | 0.4426 | 0.5216 | 0.3303 | 0.4360 |
| Glyceric acid | 0.6667 | -0.1454 | 0.5318 | 0.6051 | 0.5215 | 0.5935 |
| 4-hydroxyproline | 0.6667 | 0.1445 | 0.6494 | 0.7143 | 0.5781 | 0.6359 |
| Succinic acid | 0.5556 | -0.0848 | 0.6868 | 0.7255 | 0.6442 | 0.6746 |
| Lactic acid | 0.5556 | 0.1022 | 0.7070 | 0.7255 | 0.6746 | 0.6746 |
| Proline | 0.6667 | -0.2183 | 0.7255 | 0.7255 | 0.6578 | 0.6746 |

**Supplementary Table S5. Complete metabolite-level statistics for the diabetic versus non-diabetic comparison in plasma from empty-defect animals.** AUC values indicate ROC-based discriminatory performance. logFC was calculated as diabetic relative to non-diabetic animals; positive values indicate higher abundance in diabetic animals and negative values lower abundance. ROC p and ROC q indicate the raw and Benjamini–Hochberg-adjusted p-values from the ROC-associated unpaired two-sided Student’s t-test. DE p and DE q indicate the raw and Benjamini–Hochberg-adjusted p-values from the limma differential-abundance analysis. The comparison included non-diabetic empty-defect plasma n = 3 and diabetic empty-defect plasma n = 3.

| **Metabolite** | **AUC** | **logFC** | **ROC p** | **ROC q** | **DE p** | **DE q** |
| --- | --- | --- | --- | --- | --- | --- |
| Isoleucine | 1.0000 | -0.5230 | 0.0025 | 0.0825 | 0.0096 | 0.0844 |
| 2-aminobutyric acid | 1.0000 | -0.9593 | 0.0123 | 0.1968 | 0.0001 | 0.0023 |
| Glutamate | 1.0000 | 0.7297 | 0.0189 | 0.1968 | 0.0059 | 0.0844 |
| Uracil | 1.0000 | 0.5144 | 0.0239 | 0.1968 | 0.0102 | 0.0844 |
| Valine | 1.0000 | -0.3915 | 0.0308 | 0.1980 | 0.0896 | 0.4200 |
| Glutaric acid | 1.0000 | 0.3417 | 0.0360 | 0.1980 | 0.0722 | 0.4200 |
| Pyruvic acid | 0.9167 | -0.3061 | 0.0487 | 0.2297 | 0.1145 | 0.4200 |
| Succinic acid | 0.7500 | -0.2231 | 0.1411 | 0.5718 | 0.2827 | 0.7102 |
| Glutamine | 0.7500 | 0.1913 | 0.1559 | 0.5718 | 0.3808 | 0.7854 |
| Alanine | 0.7500 | 0.1750 | 0.1841 | 0.5796 | 0.3571 | 0.7854 |
| Cholesterol | 0.8333 | -0.3186 | 0.1932 | 0.5796 | 0.1105 | 0.4200 |
| Myo-inositol | 0.7500 | 0.2086 | 0.2147 | 0.5904 | 0.2917 | 0.7102 |
| Tryptophan | 0.8333 | 0.2273 | 0.2686 | 0.6819 | 0.3013 | 0.7102 |
| Tyrosine | 0.7500 | -0.1399 | 0.2895 | 0.6823 | 0.4653 | 0.8081 |
| Maltose | 0.5833 | 0.3403 | 0.3531 | 0.7768 | 0.1127 | 0.4200 |
| Sucrose | 0.6667 | -0.2377 | 0.3773 | 0.7782 | 0.2378 | 0.7102 |
| Glycerol | 0.5833 | -0.1457 | 0.4041 | 0.7844 | 0.4441 | 0.8081 |
| Methionine | 0.7500 | -0.1441 | 0.5014 | 0.8968 | 0.4578 | 0.8081 |
| Malic acid | 0.5000 | -0.9341 | 0.5163 | 0.8968 | 0.5659 | 0.8661 |
| Glyceric acid | 0.5000 | -0.2297 | 0.5694 | 0.9248 | 0.2885 | 0.7102 |
| Citric acid | 0.6667 | 0.1122 | 0.6127 | 0.9248 | 0.5774 | 0.8661 |
| Arabitol | 0.7500 | 0.1336 | 0.6216 | 0.9248 | 0.5021 | 0.8285 |
| Serine | 0.7500 | -0.0871 | 0.6445 | 0.9248 | 0.6592 | 0.9457 |
| Threonine | 0.5833 | -0.0729 | 0.7017 | 0.9618 | 0.7047 | 0.9689 |
| Proline | 0.5000 | 0.0631 | 0.7286 | 0.9618 | 0.7659 | 0.9898 |
| Lactic acid | 0.6667 | 0.0599 | 0.8041 | 0.9938 | 0.7821 | 0.9898 |
| Uric acid | 0.5000 | 0.0629 | 0.8322 | 0.9938 | 0.8099 | 0.9898 |
| Leucine | 0.6667 | 0.0559 | 0.9122 | 0.9938 | 0.8918 | 0.9910 |
| Urea | 0.5000 | 0.0320 | 0.9127 | 0.9938 | 0.8774 | 0.9910 |
| Glucose | 0.5833 | 0.0159 | 0.9404 | 0.9938 | 0.9371 | 0.9910 |
| Cysteine | 0.5833 | 0.0169 | 0.9476 | 0.9938 | 0.9421 | 0.9910 |
| Fumaric acid | 0.5000 | -0.0050 | 0.9817 | 0.9938 | 0.9822 | 0.9910 |
| 4-hydroxyproline | 0.5833 | 0.0030 | 0.9938 | 0.9938 | 0.9910 | 0.9910 |

**Supplementary Table S6. Complete metabolite-level statistics for the diabetic versus non-diabetic comparison in plasma from scaffold-treated animals.** AUC values indicate ROC-based discriminatory performance. logFC was calculated as diabetic relative to non-diabetic animals; positive values indicate higher abundance in diabetic animals and negative values lower abundance. ROC p and ROC q indicate the raw and Benjamini–Hochberg-adjusted p-values from the ROC-associated unpaired two-sided Student’s t-test. DE p and DE q indicate the raw and Benjamini–Hochberg-adjusted p-values from the limma differential-abundance analysis. The comparison included non-diabetic scaffold-treated plasma n = 3 and diabetic scaffold-treated plasma n = 4.

| **Metabolite** | **AUC** | **logFC** | **ROC p** | **ROC q** | **DE p** | **DE q** |
| --- | --- | --- | --- | --- | --- | --- |
| Glutamate | 0.8393 | 0.3286 | 0.0243 | 0.3733 | 0.0279 | 0.3528 |
| Fructose-6-phosphate | 0.8214 | -0.7566 | 0.0321 | 0.3733 | 0.0125 | 0.3528 |
| Pyruvic acid | 0.8750 | 0.5082 | 0.0368 | 0.3733 | 0.0194 | 0.3528 |
| Citric acid | 0.8036 | 0.2807 | 0.0457 | 0.3733 | 0.0571 | 0.3614 |
| β-alanine | 0.7679 | 0.3038 | 0.0467 | 0.3733 | 0.0480 | 0.3614 |
| Uracil | 0.7321 | 0.2366 | 0.0581 | 0.3871 | 0.0816 | 0.4431 |
| Glyceric acid | 0.7679 | -0.3684 | 0.0791 | 0.4172 | 0.0559 | 0.3614 |
| Tyrosine | 0.7679 | -0.2050 | 0.0834 | 0.4172 | 0.1258 | 0.5310 |
| Glycerol-3-phosphate | 0.7143 | -0.2411 | 0.1045 | 0.4644 | 0.1162 | 0.5310 |
| Lysine | 0.7321 | -0.3249 | 0.1350 | 0.5293 | NA | NA |
| Phenylalanine | 0.7143 | -0.2389 | 0.1456 | 0.5293 | 0.1397 | 0.5310 |
| Serine | 0.7143 | 0.2281 | 0.2162 | 0.6979 | 0.1914 | 0.6612 |
| Leucine | 0.7143 | 0.1701 | 0.2401 | 0.6979 | 0.2497 | 0.7899 |
| Threonine | 0.6786 | 0.1582 | 0.2442 | 0.6979 | 0.2702 | 0.7899 |
| Valine | 0.6429 | 0.1105 | 0.3279 | 0.7781 | 0.3898 | 0.8318 |
| Asparagine | 0.6607 | 0.1014 | 0.3572 | 0.7781 | 0.4293 | 0.8318 |
| Cholesterol | 0.6429 | 0.2337 | 0.4040 | 0.7781 | 0.3924 | 0.8318 |
| Maltose | 0.6250 | 0.0553 | 0.4090 | 0.7781 | 0.7132 | 0.9034 |
| Lactic acid | 0.6607 | -0.1284 | 0.4189 | 0.7781 | 0.4135 | 0.8318 |
| Glucose | 0.5000 | -0.2155 | 0.4306 | 0.7781 | 0.3686 | 0.8318 |
| Creatinine | 0.6250 | -0.1655 | 0.4477 | 0.7781 | 0.4151 | 0.8318 |
| Methionine | 0.6964 | 0.1277 | 0.4526 | 0.7781 | 0.4380 | 0.8318 |
| Glucuronic acid | 0.6250 | -0.0833 | 0.4680 | 0.7781 | 0.6780 | 0.9034 |
| Arabitol | 0.6607 | -0.1219 | 0.4746 | 0.7781 | 0.4597 | 0.8318 |
| Isoleucine | 0.5893 | 0.1009 | 0.5123 | 0.7781 | 0.5155 | 0.8783 |
| Hypotaurine | 0.6429 | 0.0869 | 0.5516 | 0.7781 | 0.5583 | 0.8783 |
| Fructose | 0.6161 | 0.0011 | 0.5588 | 0.7781 | NA | NA |
| Alanine | 0.5357 | 0.0774 | 0.5790 | 0.7781 | 0.6009 | 0.8783 |
| Glutamine | 0.5893 | 0.0895 | 0.5811 | 0.7781 | 0.5950 | 0.8783 |
| Uric acid | 0.5357 | 0.1391 | 0.5836 | 0.7781 | 0.5372 | 0.8783 |
| Adenosine | 0.5536 | 0.2079 | 0.6956 | 0.8975 | 0.6378 | 0.8976 |
| Fumaric acid | 0.5893 | 0.0687 | 0.7205 | 0.9006 | 0.7030 | 0.9034 |
| Tryptophan | 0.5536 | 0.0286 | 0.7576 | 0.9183 | 0.8064 | 0.9299 |
| Sucrose | 0.5179 | 0.0617 | 0.8171 | 0.9439 | 0.8089 | 0.9299 |
| Uridine | 0.5179 | 0.0379 | 0.8587 | 0.9439 | 0.8459 | 0.9299 |
| Urea | 0.5179 | -0.0473 | 0.8615 | 0.9439 | 0.7988 | 0.9299 |
| Succinic acid | 0.5357 | -0.0438 | 0.8731 | 0.9439 | 0.8565 | 0.9299 |
| Glycerol | 0.5536 | -0.0304 | 0.9039 | 0.9467 | 0.8946 | 0.9443 |
| Malic acid | 0.5179 | 0.0130 | 0.9230 | 0.9467 | 0.9294 | 0.9545 |
| 4-hydroxyproline | 0.5089 | 0.0070 | 0.9471 | 0.9471 | 0.9565 | 0.9565 |

**Supplementary Table S7. Complete metabolite-level statistics for the scaffold-treated versus empty-defect comparison in non-diabetic regenerating defects.** AUC values indicate ROC-based discriminatory performance. logFC was calculated as scaffold-treated relative to empty-defect animals; positive values indicate higher abundance in scaffold-treated defects and negative values lower abundance. ROC p and ROC q indicate the raw and Benjamini–Hochberg-adjusted p-values from the ROC-associated unpaired two-sided Student’s t-test. DE p and DE q indicate the raw and Benjamini–Hochberg-adjusted p-values from the limma differential-abundance analysis. NA indicates insufficient GC–MS detection for estimation of the corresponding statistic. The comparison included non-diabetic empty-defect n = 8 and non-diabetic scaffold-treated n = 7.

| **Metabolite** | **AUC** | **logFC** | **ROC p** | **ROC q** | **DE p** | **DE q** |
| --- | --- | --- | --- | --- | --- | --- |
| Leucine | 0.9286 | -0.4000 | 0.0015 | 0.0580 | 0.0034 | 0.0883 |
| Valine | 0.9107 | -0.3706 | 0.0044 | 0.0580 | 0.0070 | 0.0883 |
| Uracil | 0.9107 | -0.5092 | 0.0045 | 0.0580 | 0.0065 | 0.0883 |
| Citric acid | 0.9107 | 0.3425 | 0.0066 | 0.0646 | 0.0198 | 0.1253 |
| Isoleucine | 0.8750 | -0.3615 | 0.0173 | 0.0989 | 0.0189 | 0.1253 |
| Maltose | 0.8571 | -0.3998 | 0.0189 | 0.0989 | 0.0161 | 0.1253 |
| Serine | 0.7857 | -0.2707 | 0.0198 | 0.0989 | 0.0441 | 0.1676 |
| Lysine | 0.8125 | 0.2947 | 0.0203 | 0.0989 | NA | NA |
| Alanine | 0.7857 | -0.2919 | 0.0231 | 0.1003 | 0.0379 | 0.1599 |
| Cholesterol | 0.8036 | -0.6772 | 0.0301 | 0.1173 | 0.0235 | 0.1274 |
| Urea | 0.7679 | -0.3949 | 0.0490 | 0.1738 | 0.0344 | 0.1599 |
| Threonine | 0.7679 | -0.1950 | 0.0620 | 0.2015 | 0.1100 | 0.2613 |
| Lactic acid | 0.7500 | -0.3004 | 0.0929 | 0.2650 | 0.0765 | 0.2235 |
| Fumaric acid | 0.8571 | -0.2822 | 0.1053 | 0.2650 | 0.0891 | 0.2418 |
| Glutamine | 0.7857 | 0.3781 | 0.1146 | 0.2650 | 0.0762 | 0.2235 |
| Glucuronic acid | 0.7500 | 0.2314 | 0.1152 | 0.2650 | 0.1196 | 0.2662 |
| Phenylalanine | 0.7143 | -0.2310 | 0.1161 | 0.2650 | 0.1261 | 0.2662 |
| Adenosine | 0.6964 | 0.7712 | 0.1223 | 0.2650 | 0.0718 | 0.2235 |
| Glycerol-3-phosphate | 0.7321 | 0.2981 | 0.1526 | 0.3000 | 0.1665 | 0.3329 |
| Glyceric acid | 0.7857 | -0.5016 | 0.1538 | 0.3000 | 0.1060 | 0.2613 |
| Hypotaurine | 0.6964 | 0.1649 | 0.2233 | 0.4147 | 0.2399 | 0.4342 |
| Creatinine | 0.6071 | 0.2194 | 0.2401 | 0.4176 | 0.2144 | 0.4073 |
| Glycerol | 0.7143 | -0.2823 | 0.2463 | 0.4176 | 0.2986 | 0.4934 |
| Malic acid | 0.6786 | -0.2209 | 0.3221 | 0.5235 | 0.2723 | 0.4704 |
| Glutamate | 0.6071 | 0.1880 | 0.3510 | 0.5476 | 0.3121 | 0.4941 |
| Pyruvic acid | 0.5893 | 0.2376 | 0.4070 | 0.5865 | 0.3400 | 0.5168 |
| Uric acid | 0.5536 | -0.1762 | 0.4288 | 0.5865 | 0.3794 | 0.5248 |
| Uridine | 0.6250 | -0.1768 | 0.4360 | 0.5865 | 0.3867 | 0.5248 |
| Tyrosine | 0.6250 | 0.2057 | 0.4361 | 0.5865 | 0.3738 | 0.5248 |
| Sucrose | 0.5536 | -0.2233 | 0.4955 | 0.6438 | 0.4315 | 0.5654 |
| Succinic acid | 0.6071 | -0.1735 | 0.5118 | 0.6438 | 0.5404 | 0.6846 |
| β-alanine | 0.5714 | -0.0745 | 0.6762 | 0.8241 | 0.7138 | 0.8749 |
| 4-hydroxyproline | 0.5714 | 0.0355 | 0.7586 | 0.8965 | 0.7914 | 0.9147 |
| Methionine | 0.5714 | -0.0782 | 0.8190 | 0.9207 | 0.8118 | 0.9147 |
| Arabitol | 0.5536 | 0.0487 | 0.8312 | 0.9207 | 0.8184 | 0.9147 |
| Tryptophan | 0.5536 | 0.0243 | 0.8499 | 0.9207 | 0.8596 | 0.9296 |
| Asparagine | 0.5625 | 0.0293 | 0.9114 | 0.9558 | 0.8807 | 0.9296 |
| Glucose | 0.5536 | 0.0112 | 0.9313 | 0.9558 | 0.9353 | 0.9606 |
| Fructose-6-phosphate | 0.5536 | -0.0122 | 0.9704 | 0.9704 | 0.9668 | 0.9668 |

**Supplementary Table S8. Complete metabolite-level statistics for the scaffold-treated versus empty-defect comparison in diabetic regenerating defects.** AUC values indicate ROC-based discriminatory performance. logFC was calculated as scaffold-treated relative to empty-defect animals; positive values indicate higher abundance in scaffold-treated defects and negative values lower abundance. ROC p and ROC q indicate the raw and Benjamini–Hochberg-adjusted p-values from the ROC-associated unpaired two-sided Student’s t-test. DE p and DE q indicate the raw and Benjamini–Hochberg-adjusted p-values from the limma differential-abundance analysis. NA indicates insufficient GC–MS detection for estimation of the corresponding statistic. The comparison included diabetic empty-defect n = 8 and diabetic scaffold-treated n = 7.

**Supplementary Table S9. Complete metabolite-level statistics for the scaffold-treated versus empty-defect comparison in plasma from non-diabetic animals.** AUC values indicate ROC-based discriminatory performance. logFC was calculated as scaffold-treated relative to empty-defect animals; positive values indicate higher abundance in scaffold-treated animals and negative values lower abundance. ROC p and ROC q indicate the raw and Benjamini–Hochberg-adjusted p-values from the ROC-associated unpaired two-sided Student’s t-test. DE p and DE q indicate the raw and Benjamini–Hochberg-adjusted p-values from the limma differential-abundance analysis. The comparison included non-diabetic empty-defect plasma n = 3 and non-diabetic scaffold-treated plasma n = 3.

| **Metabolite** | **AUC** | **logFC** | **ROC p** | **ROC q** | **DE p** | **DE q** |
| --- | --- | --- | --- | --- | --- | --- |
| Glutaric acid | 1.0000 | -0.7679 | 0.0002 | 0.0070 | 0.0003 | 0.0093 |
| Arabitol | 1.0000 | -0.5172 | 0.0685 | 0.7750 | 0.0340 | 0.3739 |
| Malic acid | 1.0000 | -0.5621 | 0.0705 | 0.7750 | 0.0310 | 0.3739 |
| Myo-inositol | 1.0000 | -0.4549 | 0.1303 | 0.8427 | 0.0705 | 0.5818 |
| Isoleucine | 0.7778 | 0.0829 | 0.2151 | 0.8427 | 0.4625 | 0.8480 |
| Tryptophan | 0.7778 | 0.2386 | 0.2758 | 0.8427 | 0.2238 | 0.8114 |
| Sucrose | 0.7778 | -0.5645 | 0.3102 | 0.8427 | 0.2023 | 0.8114 |
| Citric acid | 0.7778 | -0.4164 | 0.3634 | 0.8427 | 0.2569 | 0.8114 |
| Uracil | 0.6667 | -0.1505 | 0.3637 | 0.8427 | 0.3688 | 0.8114 |
| Glucose | 0.7778 | -0.1984 | 0.3683 | 0.8427 | 0.3137 | 0.8114 |
| Maltose | 0.6667 | -0.6037 | 0.3858 | 0.8427 | 0.2647 | 0.8114 |
| 2-aminobutyric acid | 0.6667 | 0.2459 | 0.3940 | 0.8427 | 0.4489 | 0.8480 |
| Succinic acid | 0.6667 | 0.1878 | 0.4032 | 0.8427 | 0.3514 | 0.8114 |
| Fumaric acid | 0.6667 | -0.2616 | 0.4365 | 0.8427 | 0.3634 | 0.8114 |
| Uric acid | 0.6667 | 0.3033 | 0.4425 | 0.8427 | 0.3407 | 0.8114 |
| 4-hydroxyproline | 0.7778 | -0.2727 | 0.4585 | 0.8427 | 0.3672 | 0.8114 |
| Leucine | 0.6667 | 0.4166 | 0.4714 | 0.8427 | 0.4447 | 0.8480 |
| Glutamine | 0.5556 | 0.3495 | 0.4748 | 0.8427 | 0.3673 | 0.8114 |
| Cholesterol | 0.6667 | -0.1421 | 0.5544 | 0.8427 | 0.5207 | 0.8916 |
| Tyrosine | 0.7778 | 0.0744 | 0.5915 | 0.8427 | 0.6094 | 0.8916 |
| Urea | 0.6667 | -0.1215 | 0.5988 | 0.8427 | 0.5536 | 0.8916 |
| Threonine | 0.6667 | -0.0713 | 0.6013 | 0.8427 | 0.6214 | 0.8916 |
| Pyruvic acid | 0.6667 | -0.0395 | 0.6108 | 0.8427 | 0.7332 | 0.8962 |
| Glyceric acid | 0.5556 | -0.0920 | 0.6128 | 0.8427 | 0.6063 | 0.8916 |
| Valine | 0.6667 | -0.0129 | 0.6831 | 0.8876 | 0.9009 | 0.9535 |
| Glycerol | 0.6667 | -0.0705 | 0.6993 | 0.8876 | 0.6815 | 0.8962 |
| Cysteine | 0.6667 | -0.0814 | 0.7624 | 0.9009 | 0.7297 | 0.8962 |
| Lactic acid | 0.5556 | -0.0982 | 0.7644 | 0.9009 | 0.7175 | 0.8962 |
| Serine | 0.5556 | 0.0170 | 0.8137 | 0.9259 | 0.8825 | 0.9535 |
| Methionine | 0.6667 | 0.0259 | 0.8610 | 0.9471 | 0.8634 | 0.9535 |
| Glutamate | 0.5556 | -0.0297 | 0.8909 | 0.9484 | 0.8790 | 0.9535 |
| Proline | 0.5556 | -0.0096 | 0.9477 | 0.9495 | 0.9535 | 0.9535 |
| Alanine | 0.5556 | 0.0108 | 0.9495 | 0.9495 | 0.9503 | 0.9535 |

| **Metabolite** | **AUC** | **logFC** | **ROC p** | **ROC q** | **DE p** | **DE q** |
| --- | --- | --- | --- | --- | --- | --- |
| Isoleucine | 1.0000 | -0.2907 | 0.0236 | 0.5440 | 0.1374 | 0.7870 |
| Fumaric acid | 1.0000 | 0.2704 | 0.0474 | 0.5440 | 0.0618 | 0.7870 |
| Valine | 1.0000 | -0.3447 | 0.0495 | 0.5440 | 0.1213 | 0.7870 |
| Tyrosine | 0.9167 | -0.2688 | 0.0988 | 0.8153 | 0.0841 | 0.7870 |
| Glycerol | 0.8333 | 0.1857 | 0.1237 | 0.8163 | 0.2516 | 0.8002 |
| Alanine | 0.8333 | -0.1656 | 0.1722 | 0.8275 | 0.2310 | 0.8002 |
| Cysteine | 0.9167 | 0.2827 | 0.2022 | 0.8275 | 0.1237 | 0.7870 |
| Cholesterol | 0.7500 | 0.1834 | 0.2593 | 0.8275 | 0.3023 | 0.8002 |
| 4-hydroxyproline | 0.7500 | -0.4142 | 0.2792 | 0.8275 | 0.1431 | 0.7870 |
| Glutamine | 0.7500 | 0.0529 | 0.3267 | 0.8275 | 0.6854 | 0.8560 |
| Pyruvic acid | 0.6667 | -0.1453 | 0.3657 | 0.8275 | 0.4247 | 0.8002 |
| Malic acid | 0.7500 | -1.2969 | 0.3770 | 0.8275 | 0.3540 | 0.8002 |
| Serine | 0.7500 | 0.1701 | 0.3771 | 0.8275 | 0.3261 | 0.8002 |
| Maltose | 0.6667 | 0.2109 | 0.4220 | 0.8275 | 0.3198 | 0.8002 |
| Lactic acid | 0.6667 | -0.1405 | 0.4652 | 0.8275 | 0.4263 | 0.8002 |
| Threonine | 0.5833 | 0.1231 | 0.4712 | 0.8275 | 0.4593 | 0.8002 |
| Uracil | 0.6667 | -0.1361 | 0.5167 | 0.8275 | 0.4883 | 0.8002 |
| Glutamate | 0.6667 | 0.2191 | 0.5220 | 0.8275 | 0.4585 | 0.8002 |
| Leucine | 0.5000 | -0.2822 | 0.5315 | 0.8275 | 0.4498 | 0.8002 |
| Glutaric acid | 0.6667 | 0.0839 | 0.5334 | 0.8275 | 0.5577 | 0.8002 |
| Tryptophan | 0.6667 | -0.1132 | 0.5410 | 0.8275 | 0.5383 | 0.8002 |
| Methionine | 0.6667 | 0.1035 | 0.5516 | 0.8275 | 0.5436 | 0.8002 |
| Proline | 0.5833 | 0.2718 | 0.6110 | 0.8692 | 0.4946 | 0.8002 |
| Myo-inositol | 0.5000 | 0.0856 | 0.6697 | 0.8692 | 0.6375 | 0.8560 |
| Glyceric acid | 0.5000 | -0.1762 | 0.6726 | 0.8692 | 0.5484 | 0.8002 |
| Succinic acid | 0.5000 | 0.0496 | 0.7004 | 0.8692 | 0.7263 | 0.8560 |
| Sucrose | 0.5833 | -0.1024 | 0.7112 | 0.8692 | 0.6570 | 0.8560 |
| Arabitol | 0.5000 | -0.0728 | 0.7808 | 0.9054 | 0.7214 | 0.8560 |
| Urea | 0.7500 | 0.0610 | 0.7957 | 0.9054 | 0.7694 | 0.8756 |
| Uric acid | 0.5000 | -0.0209 | 0.9216 | 0.9977 | 0.9159 | 0.9979 |
| Citric acid | 0.5000 | 0.0044 | 0.9760 | 0.9977 | 0.9764 | 0.9979 |
| 2-aminobutyric acid | 0.5000 | 0.0050 | 0.9796 | 0.9977 | 0.9803 | 0.9979 |
| Glucose | 0.5000 | 0.0005 | 0.9977 | 0.9977 | 0.9979 | 0.9979 |

**Supplementary Table S10. Complete metabolite-level statistics for the scaffold-treated versus empty-defect comparison in plasma from diabetic animals.** AUC values indicate ROC-based discriminatory performance. logFC was calculated as scaffold-treated relative to empty-defect animals; positive values indicate higher abundance in scaffold-treated animals and negative values lower abundance. ROC p and ROC q indicate the raw and Benjamini–Hochberg-adjusted p-values from the ROC-associated unpaired two-sided Student’s t-test. DE p and DE q indicate the raw and Benjamini–Hochberg-adjusted p-values from the limma differential-abundance analysis. The comparison included diabetic empty-defect plasma n = 3 and diabetic scaffold-treated plasma n = 4.
